# DNA Ligases I and III Cooperate in Alternative Non-Homologous End-Joining in Vertebrates

**DOI:** 10.1371/journal.pone.0059505

**Published:** 2013-03-28

**Authors:** Katja Paul, Minli Wang, Emil Mladenov, Alena Bencsik-Theilen, Theresa Bednar, Wenqi Wu, Hiroshi Arakawa, George Iliakis

**Affiliations:** 1 Institute of Medical Radiation Biology, University of Duisburg-Essen Medical School, Essen, Germany; 2 Institute for Radiocytogenetics, Helmholtz Zentrum München, German Research Center for Environmental Health, Neuherberg, Germany; Tulane University Health Sciences Center, United States of America

## Abstract

Biochemical and genetic studies suggest that vertebrates remove double-strand breaks (DSBs) from their genomes predominantly by two non-homologous end joining (NHEJ) pathways. While canonical NHEJ depends on the well characterized activities of DNA-dependent protein kinase (DNA-PK) and LIG4/XRCC4/XLF complexes, the activities and the mechanisms of the alternative, backup NHEJ are less well characterized. Notably, the contribution of LIG1 to alternative NHEJ remains conjectural and although biochemical, cytogenetic and genetic experiments implicate LIG3, this contribution has not been formally demonstrated. Here, we take advantage of the powerful genetics of the DT40 chicken B-cell system to delineate the roles of LIG1 and LIG3 in alternative NHEJ. Our results expand the functions of LIG1 to alternative NHEJ and demonstrate a remarkable ability for LIG3 to backup DSB repair by NHEJ in addition to its essential function in the mitochondria. Together with results on DNA replication, these observations uncover a remarkable and previously unappreciated functional flexibility and interchangeability between LIG1 and LIG3.

## Introduction

In higher eukaryotes, DNA double strand breaks (DSBs) are predominantly repaired by a simple end joining process mediated by ligation that operates without homology requirements and is therefore termed non-homologous end joining (NHEJ) [1], [2], [3]. The main task of NHEJ is the restoration of structural integrity in broken DNA molecules, as it has no build-in mechanisms ensuring the preservation of DNA sequence at the break. As a consequence, NHEJ is associated with additions or deletions of nucleotides at the junction that alter the genome leaving “scars” behind [1]. Sequence preservation, when it occurs, is fortuitous and observed only for certain types of “clean” DNA ends generated by restriction endonucleases - it is unlikely for the chemically complex, modified ends generated by ionizing radiation (IR).

The dominance of NHEJ in DSB processing that manifests in higher eukaryotes coincides with the evolutionary appearance of DNA-PKcs [4]. Likely, DNA-PKcs optimized the functions of pre-existing DNA end joining factors - mainly the orthologs of KU, DNA ligase IV (LIG4) as well as of polymerases μ and λ, in bacteria and yeast - to generate a highly efficient mechanism capable of sealing, with half times of only a few minutes, large numbers of DSBs [5]. The NHEJ pathway that evolved in this way is frequently referred to as classical or canonical (C-NHEJ) to distinguish it from other repair pathways operating on similar principles (see below) [1], [2], [3]. We opt for the term DNA-PKcs-dependent (D-NHEJ) for this pathway to emphasize the significance of this kinase in its evolutionary development [2], [6].

D-NHEJ starts with the recognition and binding to the DNA ends of KU. DNA-bound KU recruits and activates DNA-PKcs, which in turn phosphorylates numerous proteins including most components of D-NHEJ and DNA-PKcs itself (see ref [1], [2], [3] for reviews). The latter autophosphorylation releases DNA-PKcs from the DNA ends and facilitates their modification by DNA end-processing activities such as Artemis, and PNK, as well as the addition of nucleotides by DNA polymerases μ and λ. Ligation is the final step in this process, occurs independently in the two DNA strands, in an iterative manner, and is catalyzed by the LIG4/XRCC4/XLF complex [1]. LIG4 is dedicated to this repair pathway and there are no known functions for this ligase outside this process.

Higher eukaryotic cells with mutations in components of D-NHEJ show defects in the rejoining of IR induced DSBs, as well as of DSBs generated during class switch recombination, by restriction endonucleases, or V(D)J recombination [1], [2], [3]. Despite this defect and under most circumstances, cells rejoin the majority of DSBs using an alternative form of NHEJ (frequently also called A-NHEJ). For DSBs induced by IR, this alternative form of NHEJ is globally suppressed by D-NHEJ [7] coming to the fore mainly when D-NHEJ is compromised - chemically or genetically [5]. Hence, it appears to operate as backup and will therefore call it here B-NHEJ [2], [8].

B-NHEJ likely operates in wild-type cells as well, when D-NHEJ fails to engage to, or to successfully process a particular DSB [2], [8]. B-NHEJ has slower kinetics and is also frequently associated with the generation of chromosome abnormalities such as deletions, translocations, inversions and other complex rearrangements [9], [10], [11], [12], [13]. When studied in defined systems, such alternative pathways of end joining frequently utilize 2–25 bp of homologous sequence (microhomology) to facilitate the alignment of broken ends [3]. Although the resulting microhomology at the junction is frequently taken as convenient diagnostic marker for the operation of this repair pathway, it does not reflect a functional requirement of B-NHEJ and is also generated/utilized, albeit infrequently, by D-NHEJ [1], [2].

It remains a matter of debate whether B-NHEJ is a single pathway or whether it reflects the functions of multiple DSB repair pathways that can be distinguished genetically and biochemically [1], [3]. As a result, its enzymology is poorly defined although activities such as PARP1, MRE11, NBS1 and CtIP have been implicated in its function. Considering that LIG4 is exclusively involved in D-NHEJ, the final ligation step in B-NHEJ must be mediated by one of the remaining DNA ligases, LIG1 or LIG3. While indirect evidence exists for the function of these ligases in B-NHEJ [13], [14], [15], [16], [17], a definitive and direct demonstration of their contribution to DSB repair is lacking, and their balance and interplay remain uncharacterized in the cellular setting.

Here, we use the chicken B cell line, DT40, and powerful conditional targeting approaches to generate mutants allowing investigations on the involvement and contribution of LIG1 and LIG3 to B-NHEJ [18].

## Results

### A Mono-ligase DT40 Mutant Shows the Function of LIG3 in DSB Repair

For a conclusive elucidation of the function of LIG1 and LIG3 to DSB repair, we generated a set of knockout and knock-in mutants in chicken DT40 cells [18]. This set of mutants (summarized in Table S1) includes, in addition to single knockouts for all three DNA ligases, also double and triple ligase knockout mutants, as well as ligase knock-in mutants in appropriately selected DNA ligase genetic background.

**Figure 1:**
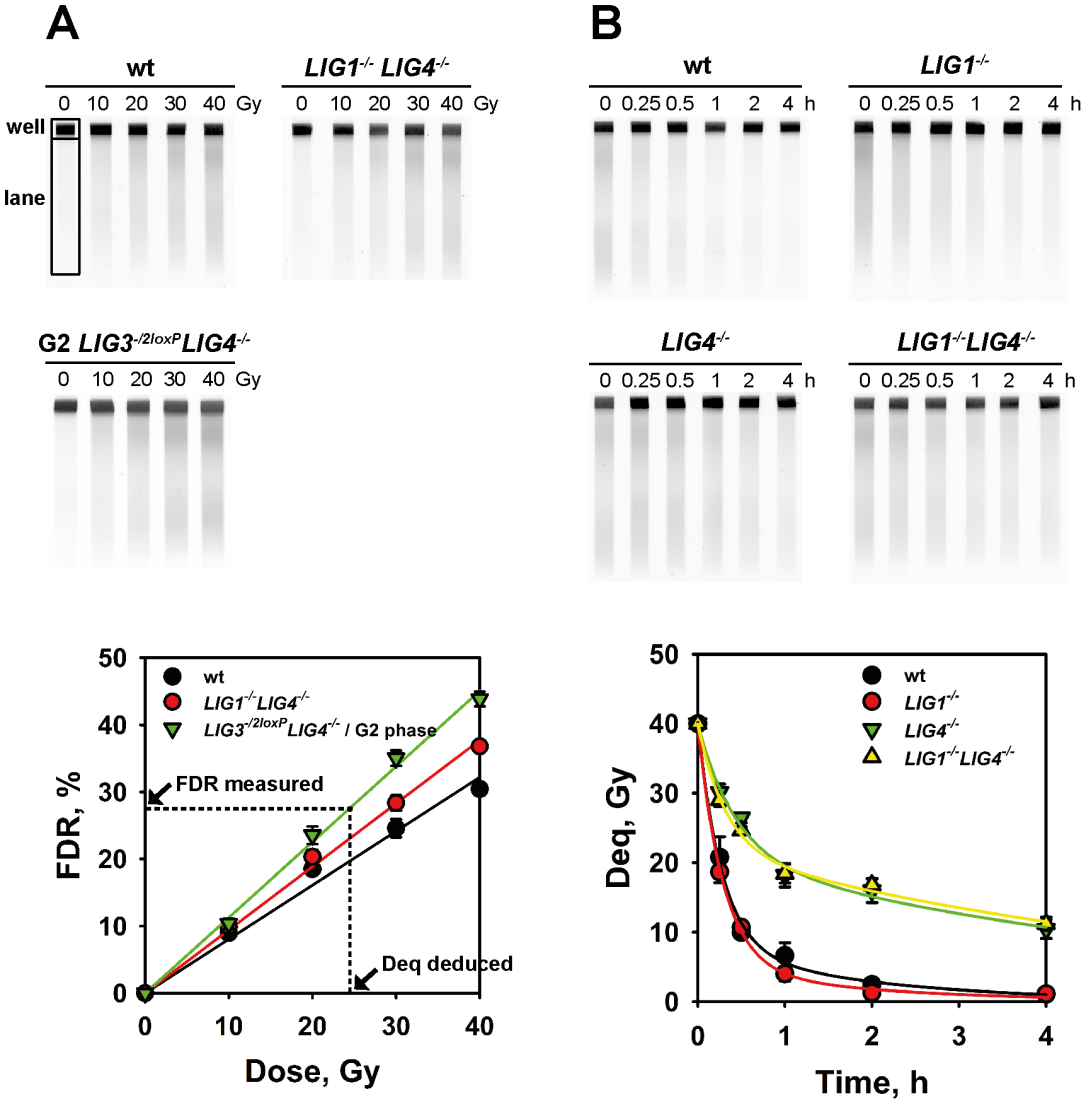
LIG3 processes IR-induced DSBs in LIG1 and LIG4 deficient cells. (**A**) Representative dose response curves for the induction of DSBs, as measured by PFGE, in cells exposed to increasing doses of X-rays. Images of ethidium bromide stained gels (upper panel) were analyzed to estimate the fraction of DNA released (FDR) from the well into the lane (regions defined as indicated) that is plotted as a function of IR dose for the indicated mutants (lower panel). Results from three independent experiments with 3 samples each were used to calculate the indicated means and standard errors. The dotted lines indicate the approach used to deduce Deq from FDR in DSB repair experiments (see text for details). (**B**) Repair kinetics of IR-induced DSBs in asynchronous wt, *LIG1^−/−^*, *LIG4^−/−^*, and *LIG1^−/−^LIG4^−/−^* cells after exposure to 40 Gy X-rays. The upper panel shows typical gels used to calculate the FDR at each repair time point, which was subsequently converted to Deq with the help of dose response curves such as those shown in A but generated with the same cell population used in the repair experiment (see text for details). Results of three determinations from at least two independent experiments were used to calculate the indicated means and standard errors (lower panel).

Exposure of wild-type (wt) DT40 cells to IR induces DSBs that fragment the DNA and cause its size–dependent migration in agarose gels under the influence of a pulsed electric field (Fig. 1A). The resulting nearly linear increase in the fraction of DNA released (FDR) from the well into the lane (see upper panel of Fig. 1A for a definition of the corresponding regions) as a function of the applied radiation dose approximates the known linear induction of DSBs with radiation dose. Dose-response curves obtained in this way vary among the different mutants (Fig. 1A) and additional variations are expected when analyzing cells in specific phases of the cell cycle [19] (see also the results with G2-phase cells in Fig. 1A). To compensate for such differences and facilitate comparisons of the repair kinetics among mutants, or with populations enriched with cells in specific phases of the cell cycle (see below), we will show here repair kinetics as Deq, instead of FDR, *versus* time plots. Deq is the equivalent dose for remaining DSBs calculated from the FDR after each repair time interval on the basis of a dose-response curve obtained from the same population of cells processed immediately after irradiation. An example of the procedure employed for each cell line and each time point is depicted by the dotted lines in Fig. 1A.

Wt DT40 cells efficiently repair IR-induced DSBs (Fig. 1B). Indeed, nearly ninety percent of those induced by 40 Gy of X-rays are processed with fast kinetics within 1 h, while the remaining 10% are processed with slower kinetics during the following 4 h of incubation. Deletion of LIG1 has no detectable effect on viability and DT40 cell growth [18]. The results in Fig. 1B show that deletion of LIG1 has no detectable consequences for the processing of DSBs either. Thus, in DT40, LIG1 is dispensable not only for semi-conservative DNA replication [18] but also for DSB repair.

As expected, deletion of LIG4 in DT40 generates a mutant with a pronounced defect in DSB repair caused by the resulting inhibition of D-NHEJ (Fig. 1B). Yet, robust residual DNA repair activity removes nearly 75% of the DSBs within 4 h, albeit with slower kinetics. This repair activity must be supported either by LIG1 or LIG3, the only remaining DNA ligases in the *LIG4* ^−/−^ mutant. Notably, deletion of LIG1 on a *LIG4* ^−/−^ genetic background generates a viable mutant with normal proliferation characteristics [18] and no DSB processing defects beyond those associated with the deletion of LIG4 (Fig. 1B). Processing of DSBs in *LIG1^−/−^LIG4^−/−^* cells must be supported by the only remaining DNA ligase, LIG3.

The robust DSB repair detected in LIG4 deficient DT40 cells may reflect either the function of alternative pathways of NHEJ or of homologous recombination repair (HRR). We have previously shown that in D-NHEJ deficient vertebrate cells, including DT40, DSB repair activity measured by PFGE mainly reflects B-NHEJ [20], [21], [22], [23]. This was the case even in a *RAD54^−/−^ KU70^−/−^* double knockout mutant [20], [21], [22], [23]. We conclude, therefore, that under the experimental conditions employed here, DSB repair in *LIG1^−/−^LIG4* ^−/−^ cells mainly reflects B-NHEJ and that LIG3 supports this form of DSB processing.

**Figure 2:**
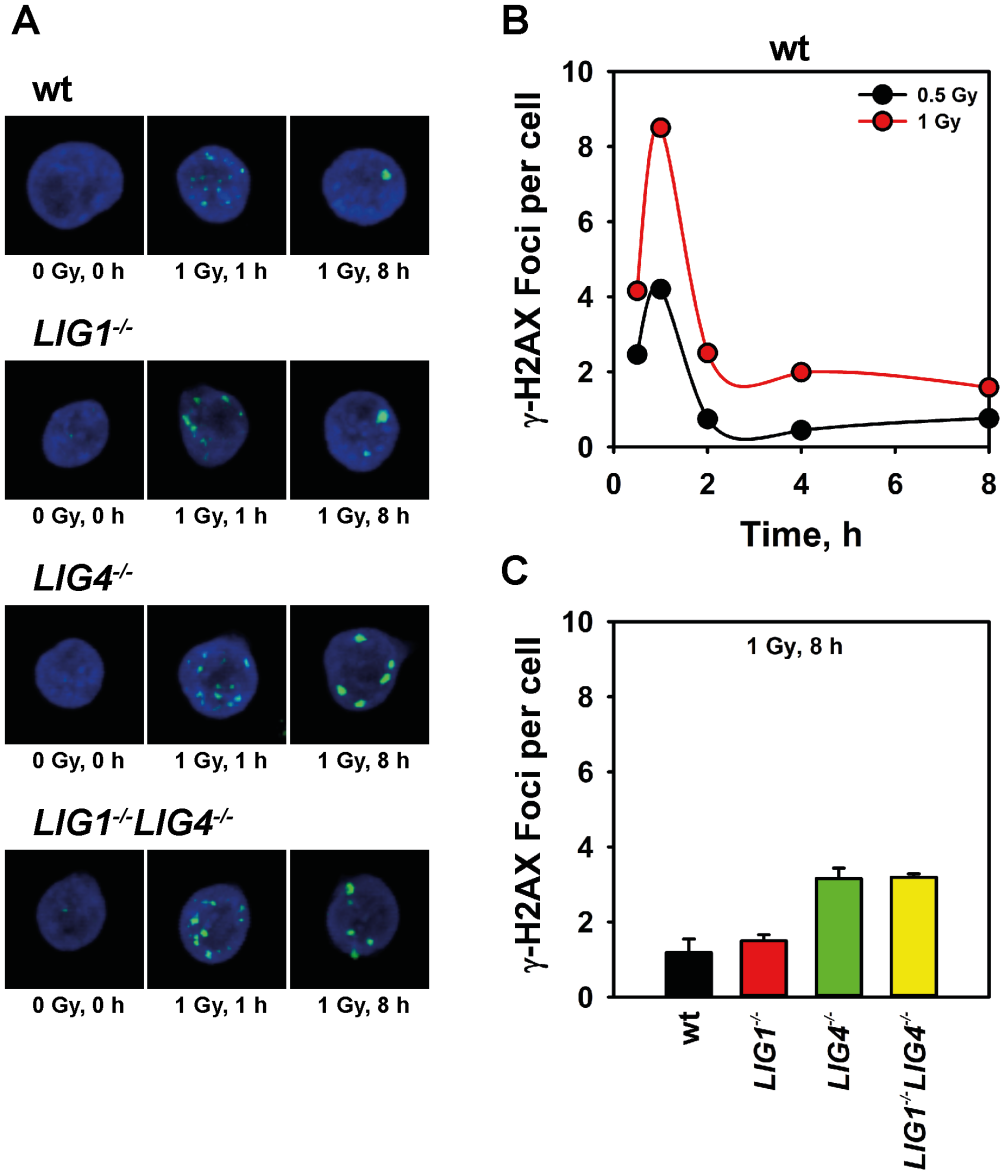
LIG3 supports processing of DSBs also after low doses of radiation. (**A**) Representative images of γ-H2AX foci formation in wt, *LIG1^−/−^*, *LIG4^−/−^* and *LIG1^−/−^LIG4^−/−^* DT40 cells after exposure to 1 Gy X-rays at the indicated times after IR. (**B**) Representative kinetics of γ-H2A.X foci formation and decay of wt DT40 cells as measured by immunostaining after exposure to 0.5 and 1 Gy X-rays. The results shown represent the analysis of 4000 nuclei in one representative experiment. (**C**) γ-H2A.X foci scored in wt, *LIG1^−/−^*, *LIG4^−/−^* and *LIG1^−/−^LIG4^−/−^* cells 8 h after exposure to 1 Gy X-rays. Foci measured in non-irradiated cells have been subtracted. Results of two independent experiments, in which 8000 nuclei were scored, were used to calculate the indicated means and standard errors.

The above experiments were carried out at high radiation doses. To test DSB repair in these mutants at lower radiation doses, we scored γ-H2AX foci formation after exposure to 0.5 and 1 Gy X-rays (Fig. 2A). Although γ-H2AX foci track directly the development of DSB-related signaling, rather than the physical processing of a DSB [6], their formation and decay are frequently used as surrogates for the induction and repair of DSBs at low radiation doses [24].

As expected, γ-H2AX foci form efficiently in DT40 cells soon after irradiation, and decay as a function of time thereafter (Fig. 2A and 2B). In wt DT40 cells repair of DSBs at these low radiation doses is indicated by the decay of γ-H2AX foci after the initial peak at ∼1 h after IR. The number of residual γ-H2AX foci scored 8 h after exposure to 1 Gy is in *LIG1^−/−^* cells similar to the wt (Fig. 2A and 2C), suggesting, in agreement with PFGE experiments, similar DSB repair capacity in the two cell lines. Higher numbers of residual γ-H2AX foci are detected, as expected, in *LIG4* ^−/−^ cells 8 h after IR (Fig. 2A and 2C), but this repair defect is not further enhanced in the double mutant *LIG1^−/−^LIG4^−/−^*.

The above observations in aggregate suggest that independently of the dose of radiation applied, LIG3, as sole ligase, supports processing of DSBs in D-NHEJ deficient cells and that LIG1 is not required for this function.

### Conditional Down-regulation of LIG3 Reveals the Function of LIG1 in Alternative NHEJ

**Figure 3:**
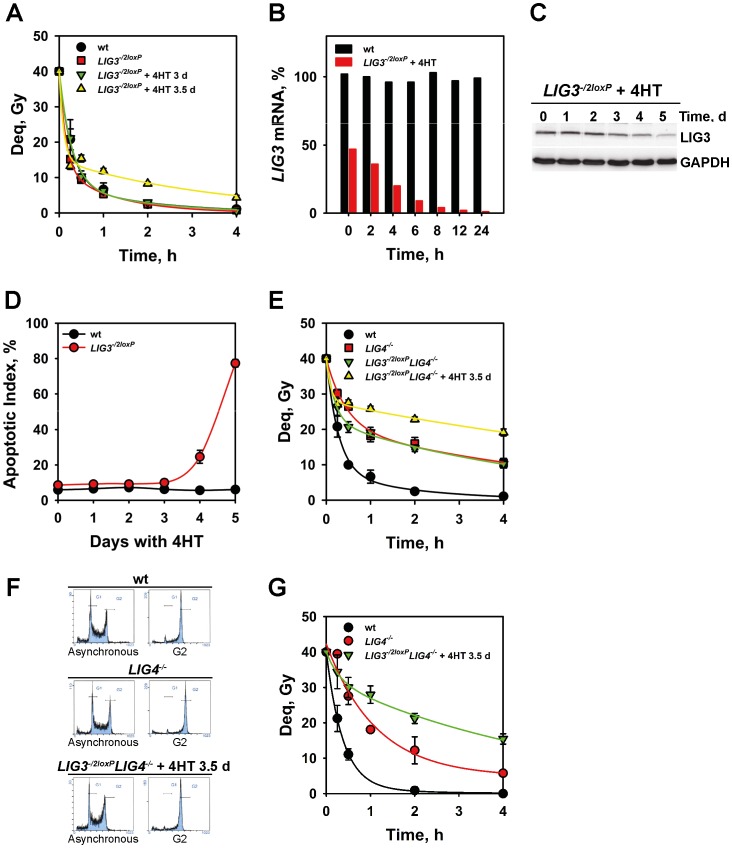
Conditional knockout of LIG3 reveals the function of LIG1 in the processing of IR-induced DSBs. (**A**) Kinetics of DSB processing in the indicated mutants after treatment with 4HT for the indicated periods of time. Other details are as in Fig. 1B. Results from three independent experiments with 3 samples each were used to calculate the indicated means and standard errors. (**B**) *LIG3* mRNA levels measured by real-time PCR in wt and *LIG3^−/2loxP^* cells after different incubation times with 4HT. The mRNA level measured in wt cells was set to 100%. (**C**) Western blot analysis of LIG3 protein in *LIG3^−/2loxP^* cells after treatment with 4HT for the indicated periods of time. A mouse monoclonal antibody against human LIG3 (clone 1F3) that recognizes the chicken LIG3 was used. GAPDH is a loading control. (**D**) Apoptotic index measured by microscopically scoring nuclear fragmentation and pycnosis in wt and *LIG3^−/2loxP^* cells at various times after treatment with 4HT. Results from two independent experiments in each of which 1000 cells were scored were used to calculate the indicated means and standard errors. (**E**) As in A for the indicated mutants. (**F**) Representative cell-cycle distribution histograms obtained by flow cytometry in wt, *LIG4^−/−^* and *LIG3^−/2loxP^LIG4^−/−^* cells treated with 4HT for 3.5 days before and after enrichment by centrifugal elutriation in G2 phase of the cell cycle. (**G**) Kinetics of DSB processing in cells enriched by centrifugal elutriation in the G2 phase of the cell cycle as shown in F.

Genetic studies on the roles and the interplay between LIG3 and LIG1 in DSB repair were hampered by the lethality of *LIG3^−/−^* cells [18], [25]. To partly overcome this limitation we generated a conditional mutant, *LIG3^−/2loxP^*. This mutant carries one null *LIG3* allele and one conditional allele with *loxP* sites inserted between several exons to allow their controlled excision by Cre recombinase. In the DT40 cells used in the present work, Cre is constitutively expressed in the cytoplasm but translocates into the nucleus after treatment with 4-hydroxytamoxifen (4HT) [18]. *LIG3^−/2loxP^* cells process DSBs with kinetics indistinguishable from that of wt cells (Fig. 3A). As a result of single allele expression, *LIG3* mRNA is in *LIG3^−/2loxP^* cells 50% reduced (Fig. 3B). We conclude that the associated reduction in LIG3 protein levels leaves unchanged the processing potential of these cells, even at radiation doses producing large numbers of DSBs.

To examine the effect on DSB processing of a further reduction in LIG3 protein level, we analyzed DSB repair kinetics in *LIG3^−/2loxP^* cells exposed to radiation after incubation with 4HT. In wt DT40, long-term treatment with 4HT leaves unchanged the distribution of cells throughout the cell cycle (Fig. S1A), their proliferative capacity (Fig. S1B), and their ability to repair DSBs (Fig. S1C). In *LIG3^−/2loxP^* cells, on the other hand, and as a direct consequence of excision of the conditional allele, 4HT causes a rapid reduction in *LIG3* mRNA to less than 10% of the controls within 6 h and to practically undetectable levels 24 h later (Fig. 3B). The reduction in mRNA causes a reduction in LIG3 protein (Fig. 3C). Notably, this reduction in protein levels is much slower than the reduction in mRNA and becomes detectable only 3 d after 4HT treatment. Depletion of LIG3 is lethal in DT40 [18] and causes apoptosis starting 4 days after treatment with 4HT (Fig. 3D).

As protein level should determine protein function in DSB repair, we studied in greater detail the significance of the slow kinetics of LIG3 protein decay shown in Fig. 3C. Since apoptosis in 4HT treated *LIG3^−/2loxP^* compromises reliable analysis of protein levels after long incubation times (Fig. 3D), we analyzed LIG3 levels in *LIG3^−/2loxP^Cdc9* cells (see below for a more detailed description of these cells). In this mutant, treatment with 4HT deletes LIG3 without inducing cell lethality because mitochondria function is rescued by the yeast *LIG1* homolog, Cdc9 [18]. Treatment of these cells with 4HT causes a clear reduction in LIG3 level, which further validates our conditional knockout system. However, here again the LIG3 polypeptide remains detectable even 10 d after 4HT treatment further documenting its unusual stability in DT40 (Fig. S2A). The stability of LIG3 is also indicated by the lack of any reduction 8 h after treatment with cycloheximide (Fig. S2B); longer incubations caused apoptosis and were therefore excluded from the analysis. On the other hand, the same treatment shows clear reduction in Rad51 levels, similar to that reported earlier [26]. Thus, the LIG3 protein appears to display an unusual and hitherto not explainable stability in DT40.

For the interpretation of the DSB repair results, residual LIG3 activity rather than residual LIG3 protein is the relevant parameter. Therefore, we used different, independent methods to evaluate LIG3 protein activity. Notably, results summarized below show no detectable, residual LIG3 activity in extracts of *LIG3^−/2loxP^* cells, as measured by an *in vitro* DNA end-joining assay, 3 d after incubation with 4HT. Also, extracts of *LIG3^−/2loxP^Cdc9* cells, tested with the same assay, show a practically complete loss of LIG3 activity already 2 d after treatment with 4HT (Fig. S2C). To confirm that DNA end-joining activity loss in extracts of 4HT-treated *LIG3^−/2loxP^* cells is caused by LIG3 activity loss, we carried out an activity-rescue experiment. The results summarized in (Fig. S2D) show convincingly the recovery of *in vitro* DNA end joining after addition in extracts of 4HT-treated (2 d) *LIG3^−/2loxP^* cells, 5 ng of purified LIG3β. Notably, the same results also document that LIG3 activity cannot be detected in 4HT-treated *LIG3^−/2loxP^* cells even after a tenfold increase in extract amount (from 0.5 to 5 µg) and that the end-joining capacity of extracts containing LIG3 is not overly enhanced by addition of recombinant LIG3 (Fig. S2D).

Finally, rapid loss of LIG3 activity is also documented in extracts of 4HT-treated *LIG3^−/2loxP^* cells using a biochemical assay measuring the ligation of oligo-dTs annealed on poly-dA (Fig. S2E). Evidently, this assay also detects primarily the activity of LIG3 and nearly 80% activity loss is measured two days after incubation with 4HT. In aggregate, the above results demonstrate that upon treatment with 4HT, *LIG3^−/2loxP^* cells loose LIG3 activity within 2–3 days although the LIG3 protein, for unknown reasons, remains detectable by western blotting for longer periods of time.

Based on these activity results, analysis of the contribution of LIG3 to DSB repair in *LIG3^−/2loxP^* cells was carried out 3 or 3.5 d after incubation with 4HT – a treatment duration expected to practically achieve complete loss of LIG3 activity.

*LIG3^−/2loxP^* cells treated with 4HT for 3d show normal processing of DSBs (Fig. 3A). When the same cells are tested 3.5 d after treatment, a detectable reduction in DSB processing is observed increasing the proportion of DSBs that are repaired with slow kinetics.

In the 4HT-treated *LIG3^−/2loxP^* mutant, DSBs are still processed predominantly by D-NHEJ utilizing LIG4. To analyze the contribution of LIG3 to DSB processing in the absence of LIG4 and to analyze the ability of LIG1 to support this function, we tested the *LIG3^−/2loxP^LIG4^−/−^* double mutant. Figure 3E shows that *LIG3^−/2loxP^LIG4^−/−^* cells process a larger proportion of DSBs with slow kinetics, as expected in the absence of LIG4, but indistinguishably from that of the *LIG4^−/−^* mutant. Further reduction of LIG3 by treatment with 4HT for 3.5 d detectably compromises DSB repair (Fig. 3E).

Together, the above results point to a contribution of LIG3 to the efficient removal of a subset of IR-induced DSBs. However, residual DSB processing removes nearly 50% of the DSBs within 4 h, and this processing must be predominantly ascribed to LIG1.

B-NHEJ operates more efficiently during the G2 phase of the cell cycle [20]. Therefore, we studied DSB processing in *LIG4^−/−^*, or 4HT-treated (3.5 d) *LIG3^−/2loxP^LIG4^−/−^* populations enriched in G2-phase cells by centrifugal elutriation (Fig. 3F). Elutriation-mediated fractionation of G2-enriched cell populations also removes apoptotic, or otherwise damaged cells, thus reducing potential artifacts in DSB detection. A reduction in the efficiency of DSB processing, slightly larger than that seen in asynchronous cells, is observed in the double mutant after treatment with 4HT supporting a role for LIG3 in the efficient processing of subsets of DSBs in the G2 phase of the cell cycle as well (Fig. 3G). However, here again, residual DSB repair activity points to the significant contribution of LIG1.

The reduction in DSB processing after depletion of LIG3 may reflect suboptimal repair by LIG1 of a small subset of DSBs that are optimally repaired only by LIG3. However, this result may also reflect other consequences of LIG3 depletion. Thus, it is possible that the higher Deq values measured after LIG3 depletion actually reflect the generation of secondary, replication-dependent DSBs produced from unrepaired SSBs, rather than reduced repair of IR generated DSBs. This is because LIG3 is implicated in the repair of SSBs [27], and its depletion may increase the generation of replication-dependent DSBs through the associated inhibition of SSB-processing. Finally, it is possible that the onset of apoptosis observed in this mutant 4 d after treatment with 4HT causes DNA degradation that is erroneously detected as reduced repair. To address these points we generated and tested additional DT40 mutants and the results obtained are presented in the following sections.

### Deletion of Nuclear LIG3 Reveals Conclusively the Function of LIG1 in Alternative NHEJ

The inception of apoptosis at low levels of LIG3 in the conditional DT40 *LIG3* mutants hampers conclusive analysis of LIG1 function in DSB repair and the investigation of a possible specific requirement for LIG3 for a subset of DSBs. To overcome this limitation we rescued lethality of *LIG3^−/−^* cells by complementing the associated mitochondria defect. Indeed, lethality of *LIG3^−/−^* mutants is rescued by the expression of diverse DNA ligases endowed with a mitochondria targeting sequence [14], [15], [18].

**Figure 4:**
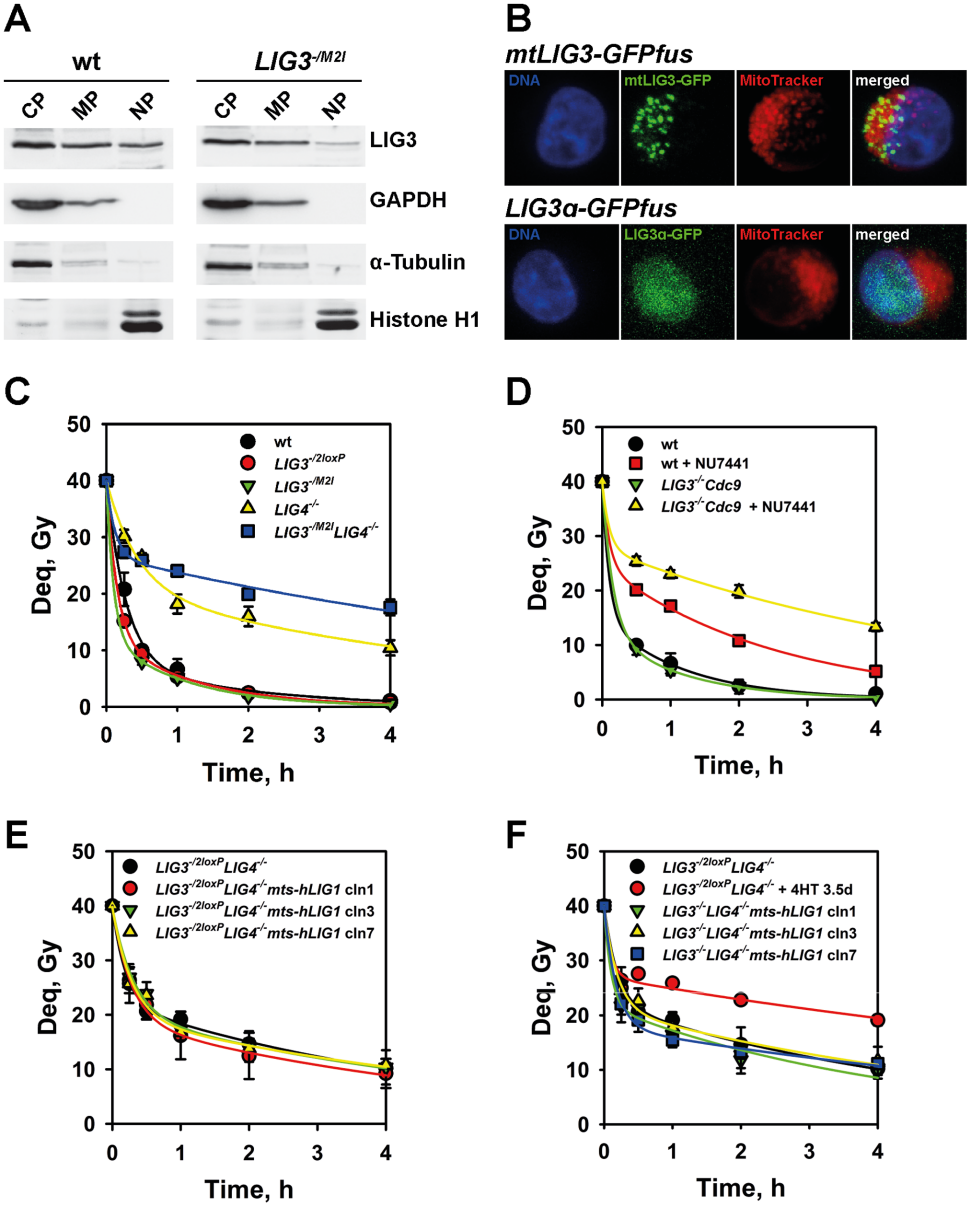
Deletion of nuclear LIG3 reveals a function for LIG1 in the processing of IR-induced DSBs. (**A**) Western blot analysis of LIG3 protein in the cytosolic fraction (CP), the mitochondria fraction (MP) and the nuclear fraction (NP) of wt and *LIG3^−/M2I^* cells. GAPDH, α-Tubulin, and histone H1 are fractionation- and loading-controls. (**B**) Subcellular localization studies by live cell imaging using mtLIG3-GFP fusion protein to follow the intracellular distribution mtLIG3 and MitoTracker Deep Red to visualize the mitochondria. Cell nuclei were counterstained with Hoechst 33342. Note the colocalization between GFP and Deep Red that indicates the localization of mtLIG3 in the mitochondria. (**C**) Kinetics of DSB repair in wt, *LIG3^−/2loxP^*, *LIG3^−/M2I^*, *LIG4^−/−^* and *LIG3^−/M2I^LIG4^−/−^* after exposure to 40 Gy X-rays. Results from at least two independent experiments with 4 samples each were used to calculate the indicated means and standard errors. Other details are as in Fig. 1B. (**D**) Kinetics of DSB repair in wt and *LIG3^−/−^Cdc9* cells measured in the presence or absence of 10 µM NU7441, a DNA-PKcs specific inhibitor. This *LIG3^−/−^* mutant is viable as a result of the expression of the yeast homolog of LIG1, Cdc9 [18]. Other details are as in C. (**E**) Kinetics of DSB repair in asynchronous *LIG3^−/2loxP^LIG4^−/−^* and clones 1, 3 and 7 of *LIG3^−/2loxP^LIG4^−/−^*mts-*hLig1* cells. Other details are as in C. (**F**) Kinetics of DSB repair in asynchronous *LIG3^−/2loxP^LIG4^−/−^*, clones 1, 3 and 7 of *LIG3^−/−^Lig4^−/−^mts*-*hLig1* cells and of *LIG3^−/2loxP^LIG4^−/−^*3.5 days after 4HT treatment, respectively. Other details are as in C.

The DT40 mutant, *LIG3^−/M2I^*, carries one inactivated *LIG3* allele and one mutated allele that lacks the second translation initiation site (M2I) normally utilized to generate the nuclear version of the enzyme [18]. As a result, *LIG3^−/M2I^* cells express exclusively the mitochondrial form of LIG3. Despite lack of nuclear LIG3, *LIG3^−/M2I^* cells are viable and grow with kinetics similar to the wt [18]. As expected, in *LIG3^−/M2I^* cells only traces of LIG3 are detectable by western blotting in the nuclear fraction of a cellular extract, whereas high amounts of the enzyme are detected in the mitochondria and the cytoplasmic fractions (Fig. 4A). Furthermore, analysis of intracellular mtLIG3 localization through expression of a GFP-tagged protein shows clear compartmentalization in the mitochondria (Fig. 4B).

Following exposure to IR, *LIG3^−/M2I^* cells show effective repair of DSBs that is indistinguishable from that of wt or *LIG3^−/2loxP^* cells (Fig. 4C). In *LIG3^−/M2I^* cells, nuclear DSB repair is mainly supported by LIG4, and LIG1. Since extensive depletion of nuclear LIG3 in this DSB repair proficient mutant, fails to detectably alter the repair kinetics, we conclude that secondary, replication-dependent DSBs possibly arising from unrepaired SSBs as a consequence of LIG3 depletion, only have a marginal effect on Deq.

To further analyze the contribution of LIG1 to DSB repair and its interplay with LIG3, we generated the double mutant *LIG3^−/M2I^LIG4^−/−^* and tested its viability and its DSB repair capacity. In this mutant, which is also viable [18], LIG1 is the only remaining nuclear DNA ligase. *LIG3^−/M2I^LIG4^−/−^* cells process DSBs (Fig. 4C) with efficiency slightly lower than *LIG4* ^−/−^ cells and similar to that seen with the corresponding conditional *LIG3* mutant after treatment with 4HT for 3.5 d (Fig. 3E). This result confirms the function of LIG1 in alternative NHEJ and provides further support for a relatively preferential function of LIG3 in a small subset of DSBs.

In the above experiment, traces of LIG3 detectable in the nucleus of *LIG3^−/M2I^* cells (Fig. 4A) and the observation that even low DNA ligase levels support efficient DSB repair [28] may be regarded as evidence that LIG3 still contributes to the DSB processing measured in this setup.

To address this point and conclusively determine the role of LIG1 in DSB repair, we devised a genetic system devoid of any form of LIG3. To this end we took advantage of our recent observation that *Cdc9,* the yeast homolog of *LIG1* that also carries a mitochondria targeting sequence, rescues the lethality associated with LIG3 depletion and allows the generation of *LIG3^−/−^* cells [18]. *LIG3^−/−^Cdc9* cells repair IR-induced DSBs with kinetics identical to wt, evidently taking advantage of LIG4 function. Since we were not successful in generating a *LIG3^−/−^LIG4^−/−^Cdc9* mutant, we used the DNA-PKcs inhibitor NU7441 to chemically compromise D-NHEJ and study the role of DT40/yeast DNA ligase I in B-NHEJ [29]. Compared to NU7441-treated wt cells, in which LIG1 and LIG3 remain active, *LIG3^−/−^Cdc9* cells show after treatment with NU7441 extensive repair of DSBs predominantly mediated by DNA ligase I (Fig. 4D). The reduced DSB repair efficiency, compared to NU7441-treated wt cells, points again to a specific role of LIG3 for a small subset of DSBs. We conclude, also in line with results presented above, that DNA ligase I supports alternative end joining of DSBs when LIG3 is absent and D-NHEJ is compromised.

To further delineate the interplay between LIG1 and LIG3 in DSB repair, we transfected hLIG1 with a mitochondrial targeting sequence (mts-hLIG1) into the *LIG3^−/2loxP^LIG4^−/−^* mutant and selected clones with stable integration of the construct. Seven clones were randomly picked and three were selected for further analysis. These clones show improved growth characteristics compared to parental cells (Fig. S3A). Real time PCR shows comparable *hLIG1* mRNA levels (Fig. S3B), whereas western blotting documents protein over-expression, albeit at different levels in the different clones (Fig. S3C). These clones carry one null and one conditional *LIG3* allele and show as expected *LIG3* mRNA levels reduced by 50% compared to the wt (Fig. S3D). Treatment of these clones with 4HT allows the generation of *LIG3^−/−^LIG4^−/−^* cells expressing DT40 LIG1 at normal levels and over-expressing mts-hLIG1. Figure S4A shows that these mutants grow as efficiently as their untreated counterparts despite the documented excision of the genomic *LIG3* segment between the *loxP* sites (Fig. S4B) and the undetectable *LIG3* mRNA (Fig. S4C). Furthermore, in these cells, the LIG3 protein is barely detectable by western blotting (Fig. S4D) and DNA end joining activity is absent from their extracts (see below) (Fig. S4E); collectively these observations confirm the LIG3 null phenotype.

Analysis of DSB processing in clones 1, 3 and 7 shows (Fig. 4E) that in *LIG3^−/2loxP^LIG4^−/−^mts-hLIG1*, ectopic expression of mts-hLIG1 does not modulate in any way DSB repair. Notably, the same clones analyzed 5 d after treatment with 4HT to delete LIG3 show DSB processing similar to the untreated controls and clearly more efficient than that measured in *LIG3^−/2loxP^LIG4^−/−^* cells 3.5 d after 4HT treatment (Fig. 4F).

In aggregate, the above observations confirm that LIG1 can support the ligation requirements of DSB processing by B-NHEJ, and show that this function depends on expression level; optimal DSB end-joining activity, similar to that achieved by LIG3, is detected when the mts-hLIG1 version of the enzyme is overexpressed – probably caused by some partition of the enzyme in the cell nucleus.

### Dominant Function of Nuclear LIG3 in Plasmid End Joining

**Figure 5:**
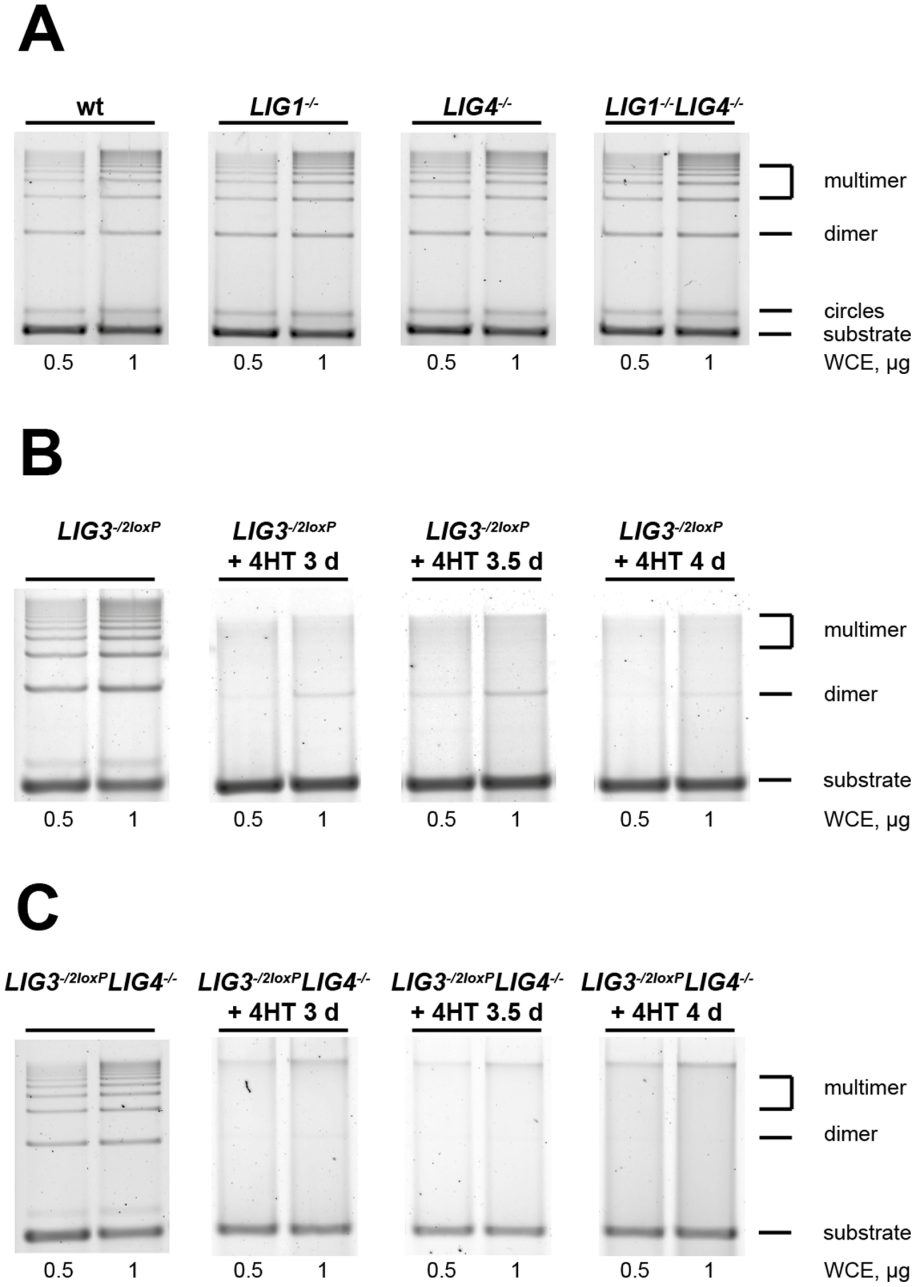
Dominant contribution of LIG3 *in-vitro* end joining. (**A**) Representative gels of *in vitro* DNA end joining of *Sal* I linearized *pSP65* plasmid using whole cell extracts of asynchronous wt, *LIG1^−/−^*, *LIG4^−/−^*, and *LIG1^−/−^LIG4^−/−^* cells. The linearized input substrate (linear) and the rejoined products (circles, dimers and multimers) generated by end joining are indicated (**B**) As in A. for *LIG3^−/2loxP^* cells after treatment with 4HT for the indicated periods of time. (**C**) As in A. for *LIG3^−/2loxP^LIG4* ^−/−^ cells after treatment with 4HT for the indicated periods of time.

Important insights into the biochemical mechanisms of DSB repair have been obtained using diverse plasmid-based *in vitro* assays. In line with earlier reports [30], [31], whole-cell extracts prepared from wt DT40 cells efficiently support the joining in *pSP65* plasmid of *Sal*I-produced DSB ends with 4 nucleotide 5′-cohesive overhangs to generate circles, dimers and multimers (Fig. 5A). The DNA end joining capacity of *LIG1^−/−^*, *LIG4* ^−/−^, and *LIG1^−/−^LIG4* ^−/−^ extracts is similar to the wt (Fig. 5A), in line with our earlier observations that DNA ligation in this assay mainly reflects LIG3 activity [30].

For further biochemical analysis of the contribution of LIG3 to plasmid DNA end joining, we prepared extracts from 4HT-treated *LIG3^−/2loxP^* cells. DNA end joining activity is barely detectable 3 d after incubation with 4HT and remains at similarly low levels up to 4 d (Fig. 5B). Since *LIG3^−/2loxP^* cells have normal levels of LIG1 and LIG4, the result confirms the dominant role of LIG3 in this version of the assay.

A reduction in plasmid DNA end joining after treatment with 4HT similar in magnitude to that measured in *LIG3^−/2loxP^* is also observed in extracts of *LIG3^−/2loxP^LIG4^−/−^* cells (Fig. 5C). Finally, there is no detectable LIG3 activity in the extracts of *LIG3^−/−^LIG4^−/−^mts-hLIG1* cells (Fig. S4E).

This biochemical data in aggregate allows us to conclude that LIG3, as a result of its amino terminal Zn-finger domain [31], [32], has a dominant contribution to DNA end joining under the reaction conditions employed.

### LIG3 and LIG1 Contribute to the Survival of IR-exposed Cells Albeit Less Efficiently than LIG4

**Figure 6:**
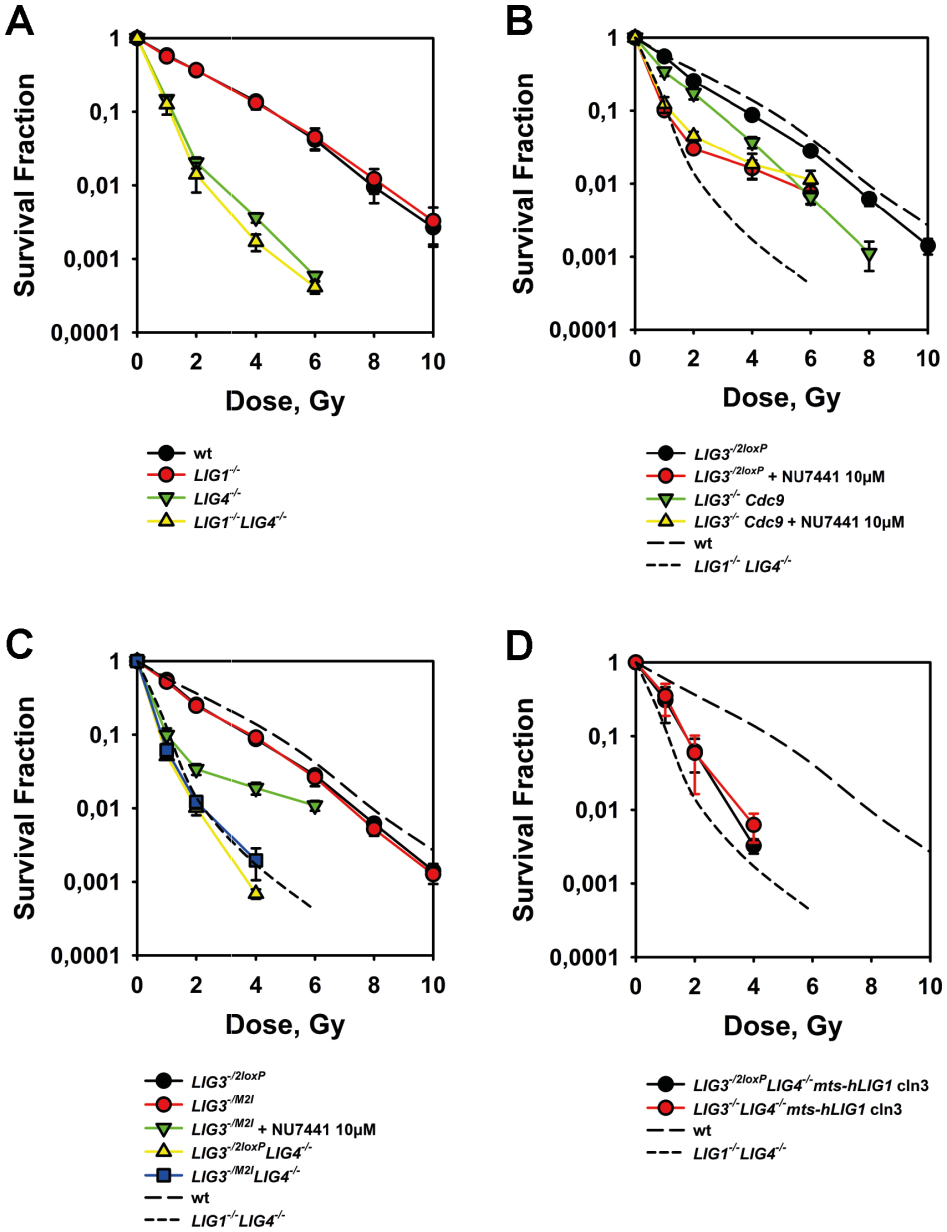
LIG1 and LIG3 contribute to the survival of cells exposed to IR. (**A**) Cell survival measured by colony formation in wt, *LIG1^−/−^*, *LIG4* ^−/−^ and *LIG1^−/−^LIG4^−/−^* cells after exposure to increasing doses of X-rays. Results from three independent experiments with 3 replicates each were used to calculate the indicated means and standard errors. (**B**) As in A. for *LIG3^−/2loxP^*, and *LIG3^−/−^Cdc9* cells after treatment for 1 h before and 4 h after IR with 10 µM NU7441. The dashed lines trace for comparison the results of wt and *LIG1^−/−^LIG4* ^−/−^ cells. (**C**) As in B. for *LIG3^−/2loxP^*, *LIG3^−/M2I^, LIG3^−/2loxP^LIG4^−/−^*, and *LIG3^−/M2I^LIG4* ^−/−^ cells. Results from at least two independent experiments with 3 replicates each were used to calculate the indicated means and standard errors. (**D**) As in B. for clone 3 of *LIG3^−/2loxP^LIG4^−/−^mts-hLIG1* and *LIG3^−/−^LIG4^−/−^mts-hLIG1.* Results from at least two independent experiments with 3 replicates each were used to calculate the indicated means and standard errors.

Exposure of wt DT40 cells to IR compromises their reproductive integrity (Fig. 6A). Deletion of LIG1 has no effect on this endpoint suggesting that LIG1 is not essential for any of the repair pathways that support the survival of irradiated DT40 cells (Fig 6A). As expected, deletion of LIG4 causes a marked radiosensitization (Fig. 6A), which is not exacerbated by a concomitant deletion of LIG1. In the latter mutant all ligation functions of the DNA metabolism including repair of all forms of radiation-induced DNA lesions are carried out by LIG3. The biphasic shape of the survival curve likely reflects pronounced differences in the radiosensitivity of cells throughout the cell cycle [33], [34].

*LIG3^−/2loxP^* cells display radiosensitivity very similar to the wt, and treatment with the DNA-PKcs specific inhibitor NU7441 increases radiosensitivity to *LIG4^−/−^* levels (the radioresistant tail likely reflects cells in radioresistant phases of the cell cycle and/or a ∼5% cell population, which, for unknown reasons, is not responding to the drug) (Fig. 6B). Notably, *LIG3^−/−^Cdc9* cells are more radiosensitive than the wt (Fig. 6B) pointing to some unique function of LIG3 in cell survival after exposure to IR, or to dominant negative effects exerted by Cdc9. However, after treatment with NU7441, *LIG3^−/−^Cdc9* cells become indistinguishable from the *LIG1* ^−/−^ *LIG4* ^−/−^ mutant (Fig. 6B) suggesting that the increased radiosensitivity derives from compromised D-NHEJ. These observations indicate that LIG1 substitutes with similar efficiency LIG3 in repair functions supporting cell survival.

*LIG3^−/M2I^* cells also show radiosensitivity to killing very similar to that of the wt and add further support to the notion that nuclear LIG3 is not essential for survival in IR exposed cells (Fig. 6C). Treatment of this mutant with NU7441 increases radiosensitivity to the same extent as in the wt or in *LIG3^−/2loxP^* cells suggesting that the reduced level of nuclear LIG3 has in the presence of LIG1 no influence on cell radiosensitivity to killing, even when D-NHEJ is compromised. The double mutants *LIG3^−/2loxP^LIG4^−/−^* and *LIG3^−/M2I^LIG4^−/−^* show radiosensitivity indistinguishable from that of the double *LIG1^−/−^LIG4^−/−^* mutant (Fig. 6C), further demonstrating that strong reduction in nuclear LIG3 leaves unchanged the ability of DT40 to deal with radiation lesions, presumably because these are processed by LIG1.

To further delineate the function of LIG1 in cell survival after exposure to IR, we examined clone 3 that expresses ectopically mts-hLIG1 either in the *LIG3^−/2loxP^LIG4^−/−^* or the *LIG3^−/−^LIG4^−/−^* genetic background (Fig. 6D). Compared to *LIG3^−/2loxP^LIG4^−/−^* cells, *LIG3^−/2loxP^LIG4^−/−^mts-hLIG1* and *LIG3^−/−^LIG4^−/−^mts-hLIG1* cells are slightly more radioresistant pointing to a small survival advantage conferred by overexpressed LIG1.

## Discussion

The results outlined above provide solid genetic proof for independent and robust function of both LIG1 and LIG3 in DSB repair via B-NHEJ. Together with our results on the ligation requirements of DT40 DNA replication [18] and results recently published in other cell systems [14], [15], [16], they confirm the restricted function of LIG4 in D-NHEJ and demonstrate a remarkable functional flexibility for LIG1 and LIG3 not only in semi-conservative DNA replication, but also in B-NHEJ. This flexibility may underpin the frequent loss and gain observed for LIG3 throughout the evolution [12].

In DT40, LIG3, as sole cellular DNA ligase, supports repair of DSBs in *LIG1* ^−/−^ *LIG4^−/−^* cells. In these mutants deletion of LIG4 compromises D-NHEJ, allowing DSB processing predominantly by B-NHEJ, as a contribution by HRR cannot be detected under the conditions employed [2]. Similarly, extensive DSB repair is observed in *LIG3^−/−^LIG4^−/−^* mutants rescued from mitochondria-mediated death by the ectopic expression of mts-hLIG1. Also in this mutant a single ligase family, LIG1, supports all ligation requirements of the DNA metabolism. In addition, when LIG4 deletion shifts DSB processing from D-NHEJ to B-NHEJ (a contribution of HRR remains undetectable in these mutants as well), LIG1 efficiently supports this function. Finally, the response of the *LIG3^−/−^Cdc9* mutant treated with NU7441 to compromise D-NHEJ is in also line with the involvement of LIG1 in B-NHEJ. Together with the similar radiosensitivity to killing, our data points to a remarkable ability of LIG1 and LIG3 to support DSB repair by B-NHEJ with similar efficiency in an apparently interchangeable manner.

The results extend and modify earlier biochemical observations [17], [31], [35], [36], [37], which favored the function of LIG3 in vertebrate B-NHEJ, although LIG1 was also implicated under certain conditions [17].

The robust function of both LIG1 and LIG3 in B-NHEJ does not exclude functional specialization deriving from the domain structure of each ligase, and the associated co-factor partnerships, that may favor processing of specific subsets of DSBs by a particular ligase. This may be especially true for IR that induces a spectrum of biochemically distinct DSBs, raising the possibility that specific types are more efficiently processed by one ligase than another. It has been suggested that LIG1 and LIG3 operate in different alternative pathways of DSB repair based on their differential involvement in the formation of chromosome translocations from nuclease induced DSBs [13].

Indeed, our results also suggest differences in the involvement of LIG1 and LIG3 in alternative end joining. Thus, while deletion of LIG1 in a *LIG4^−/−^* background has no consequences on DSB rejoining, partial depletion of LIG3 shows a small defect even in wt background. This defect increases in a *LIG4^−/−^* background and becomes more pronounced during G2, when the function of B-NHEJ is at maximum [20], [21]. Also the response of the *LIG4^−/−^LIG3^−/M2I^* mutant, or the *LIG3^−/−^Cdc9* mutant after NU7441 treatment, allows similar conclusions. Possibly, a small subset of IR-induced DSBs is processed by LIG3 more efficiently than by LIG1.

Finally, we note that overexpression of hLIG1 complements the LIG3 defect in DT40 cells and that in a mouse model system, LIG1 depletion generates a more severe DNA repair defect (DSB repair was not studied here specifically) than LIG3 depletion [14]. Thus, species-specific differences in the diverse functions of LIG1 and LIG3 are possible.

LIG3 is also implicated in the formation of chromosome translocations [13], although this preference does not seem to be a general phenomenon [16]. Also the observation that LIG3 is upregulated in CML cell lines that are positive for the BCR-ABL translocation suggests niche functions for LIG3 versus LIG1 in DNA repair [38]. Specialized functions for LIG3 are also suggested by its domain structure and particularly the amino-terminus zinc-finger domain, which enhances the DNA knick-binding affinity of the enzyme and facilitates inter-molecular ligation [31].

It is commonly thought that LIG3 operates in complex with XRCC1 [39]. However, XRCC1 deficient cells fail to show decreased usage of B-NHEJ as suggested by increased usage of microhomology [40], and recent reports uncouple the function of LIG3 from XRCC1 in several cellular functions [14], [15] including repair of DSBs during Ig heavy chain class switch recombination by alternative end joining [16]. Therefore, we postulate that LIG3 operates in DT40 B-NHEJ without a requirement for XRCC1 function. Since a XRCC1 homolog could not be identified in the known DT40 genome, genetic studies on the function of this protein in DT40 are compromised at present.

Our results show that the slight advantage afforded to DT40 B-NHEJ by LIG3 can be compensated by overexpression of the mitochondria form of hLIG1. The function of LIG1 in B-NHEJ is also supported by results obtained in yeast that lack LIG3. Thus, deletion of Dnl4 or its accessory factor Nej1 reduces alternative repair approximately by half, but does not completely eliminate it, which is consistent with a role of LIG1 in yeast alternative end joining [41], [42], [43]. In human cells, LIG1 is also involved in alternative DSB repair [17] and in quiescent cells, LIG1 rather LIG3 appears involved in the repair of diverse DNA lesions - even those induced by IR or H_2_O_2_ that should include DSBs [14]. Also results obtained using Ig class switch recombination in B cells indirectly support a role for LIG1 in alternative end joining [16].

Thus, LIG1 and LIG3 may flexibly operate in B-NHEJ, further enhancing the flexibility of this repair pathway in the selection of participating factors. The apparent plasticity in participating factor selection for B-NHEJ is in stark contrast to the rigid repertoire of factors participating in D-NHEJ and may reflect its primordial nature [5].

## Materials and Methods

### Cell Culture

DT40 cells were grown in D-MEM/F12 supplemented with 10% fetal bovine serum, 1% chicken serum, 50 µM β-mercaptoethanol at 41°C in a humidified incubator with 5% CO_2_ and were routinely maintained in the logarithmic phase of growth. Mutants analyzed here were derived from the DT40-Cre1 cell line and have been described [44], [45]. They are also summarized in Table S1.

Cells were exposed to X-rays using a Pantak X-ray machine, MXR-321, operated at 320 kV, 10 mA with a 1.6-mm Al filter, at a distance of 50 cm and a dose rate of ∼2 Gy/min.

### Pulsed-field Gel Electrophoresis (PFGE)

16×10^6^ cells were pre-treated for 1 h with 100 µM Caspase III Inhibitor (Calbiochem), and irradiated on ice. After irradiation, cells were quickly returned to 41°C for repair. After each repair time interval, cells were collected and embedded in 0.5% Agarose (InCert agarose, Bio-Rad) growth medium at a final concentration of 7×10^6^ cells/ml. Cells were lysed for 18 h at 50°C in lysis buffer (10 mM Tris, 100 mM EDTA, 50 mM NaCl, 2% *N*-laurylsarcosine, 0.2 mg/ml Protease). Subsequently, plugs were transferred to washing buffer (10 mM Tris, 100 mM EDTA, 50 mM NaCl, 37°C) for 1 h and treated for 1 h with 0.1 mg/ml RNAase A at 37°C. Induction and repair of DSBs were evaluated by asymmetric field inversion gel electrophoresis (AFIGE). AFIGE was carried out in 0.5% gels of SeaKem LE Agarose (Lonza) with 0.5 µg/ml ethidium bromide, and were run in 0.5×TBE (45 mM Tris, pH 8.2, 45 mM boric acid, 1 mM EDTA) at 8°C for 40 h. During this time, electric field cycles of 50 V (1.25 V/cm) for 900 s in the forward direction alternated with cycles of 200 V (5.0 V/cm) for 75 s in reverse direction. Gels were scanned using a fluorimager (Typhoon, GE-Healthcare) and analyzed with appropriate software (Image-Quant, GE-Healthcare). The fraction of DNA released (FDR) from the well into the lane (see Figure 1A for a graphical definition) is a measure of DSBs present. FDR measured in non-irradiated samples was subtracted from FDR measured in samples exposed to IR.

In order to facilitate the inter comparison of results obtained with different mutants, repair kinetics are not presented as FDR versus time, but rather as dose equivalent (Deq) *versus* time. Deq is calculated from FDR using dose response curves as described under “Results”.

### Immunofluorescence Microscopy

Approximately 10^6^ cells were spun for 1 min at 800 g on poly-L-lysine pretreated coverslips using a cytospin centrifuge. Alternatively, cells were collected in PBS and layered on ImmunoSelect Adhesion slides (Squarix) kept on ice and were allowed to attach for 10 min. Cells were fixed for 15 min with 2% paraformaldehyde. After washing with PBS, cells were permeabilized for 5 min in P-solution (0.5% Triton X-100 in 100 mM Tris-HCl, pH 7.4, 50 mM EDTA) and blocked with PBG solution (PBS, 0.5% BSA, 0.2% Gelatin) overnight at 4°C.

After blocking, cells were incubated with primary antibody diluted in PBG solution for 1.5 h at RT. Cells were washed three times with PBS and were incubated with a secondary antibody, diluted in blocking buffer for 1 h at RT. Cell nuclei were counterstained for 30 min with 2 µg/ml 4′,6-Diamidin-2-phenylindol (DAPI), 100 mM Tris-HCl, pH 7.4, 100 mM NaCl, 5 mM MgCl_2_, 0.05% Triton X-100) and washed once with PBS. Cells were embedded using Prolong-Gold antifade (Invitrogen). Samples were scanned using a 40× objective in an automated analysis station equipped with a fluorescence microscope (AxioImager Z2, Zeiss) and controlled by the Metafer software (MetaSystems). On average, 4000 cells per sample were scored and analyzed using the same software. For visualization the following antibodies were used: anti-γH2AX mouse monoclonal antibody (JBW301, Upstate) and anti-mouse IgG secondary antibody, conjugated with AlexaFluor 488 (Invitrogen).

### RT-PCR and Real-time RT-PCR

RNA was prepared according to the protocol of the High-Pure RNA Isolation-Kit (Roche). RNA concentration was determined with the NanoDrop spectrophotometer (Thermo Scientific) and cDNA was reverse-transcribed using Maxima® First-Strand cDNA Synthesis-Kit (Fermentas) following the protocol provided by the manufacturer. This cDNA was used for real-time PCR reactions using the LightCycler® FastStart DNA Master^PLUS^-SYBR-Green-I kit according to the protocol provided by the manufacturer (Roche). The primers used were: TBP1-F: 5′-CAGCACCAACAGTCTGTCCA-3′; TBP1-R: 5′-GGGGCTGTGGTAAGAGTCTG-3′; LIG3-F: 5′-GATGACCCCAGTTCAGCCTA-3′; LIG3-R: 5′-GTGGGCTACTTTGTGGGGAA-3′; hLIG1 F1∶5′-GAATTCTGACGCCAACATGCA-3′; hLIG1 R1∶5′-CCGTCTCTCTGCTGCTATTGGA-3′; hLIG1 F2∶5′-CAGAGGCCAGAAAGACGTG-3′; hLIG1 R2∶5′-GTCCAGGTCGGGAACCTC-3′.

### SDS-PAGE and Western Blotting

Protein gel electrophoresis under denaturating conditions was carried out using 10% polyacrylamide gels and standard procedures. For western blot analysis, proteins were transferred onto nitrocellulose membranes using an iBlot dry-transfer system (Invitrogen). Equal loading and transfer efficiency were monitored by Ponceau S staining combined with immunodetection of GAPDH. After transfer, membranes were incubated in blocking buffer (5% non-fat dry milk in 0.1% Tween-20, 150 mM NaCl, 25 mM Tris-HCl, pH 7.6) for 1–2 h at room temperature. Subsequently, membranes were incubated overnight at 4°C with primary antibody appropriately diluted in blocking buffer. After three washes for 10 min with TBS-T (0.1% Tween-20, 150 mM NaCl, 25 mM Tris-HCl, pH 7.6), membranes were incubated for 1 h with secondary antibody appropriately diluted in TBS-T. Membranes were scanned using the Odyssey infrared imaging system (Li-COR) or were developed for chemiluminescence detection by using ECL+ chemiluminescence detection kit (GE Healthcare) as recommended by the manufacturer. The following primary antibodies were used: anti-LIG3 (1F3) mouse mAb (GeneTex); anti-LIG1 (10H5) mouse mAb (Santa Cruz), anti-α-Tubulin (AA13) mouse monoclonal (Sigma Aldrich), anti-histone H1 (AE-4) mouse mAb (Acris), and anti-GAPDH mouse mAb (Millipore). The secondary antibody was anti-mouse IgG conjugated with HRP (Cell Signaling), IRDye680 and IRDye800 (Li-COR).

### Measurement of Apoptotic Index

Cells were collected by centrifugation and fixed in 70% ethanol. Fixed cells were resuspended with DAPI staining solution (0,1 M Tris, pH 7.0, 0.1 M NaCl, 5 mM MgCl_2_, 0,05% Triton X-100, 2 µg/ml DAPI). After incubation for 5 min, 20 µl were analysed under a fluorescent microscope by counting the fraction of fragmented and pycnotic nuclei in 1000 cells.

### Analysis of Cell Cycle Distribution by Flow Cytometry

Measurements of cell cycle distribution were carried out with an Epics XL-MCL (Beckman-Coulter) flow cytometer. Cells were collected by centrifugation and fixed in 70% cold ethanol. Fixed cells were stained with PBS containing 40 µg/ml propidium iodide and 62 µg/ml RNaseA for 30 min at 37°C. Approximately 20,000 cells were measured and fractions of cells in different phases of the cell cycle were calculated using the Wincycle® software (Phoenix Inc.).

### Cell Fractionation in Different Phases of the Cell Cycle by Centrifugal Elutriation

About 2×10^8^ exponentially growing cells were collected and elutriated at 4°C using a Beckman JE-6 elutriation rotor and a Beckman J2-21M high-speed centrifuge at 25 ml/min (Beckman, Krefeld, Germany). Cells were loaded at 4,500 rpm, and 250 ml fractions were collected between 3,200 and 2,300 rpm at 100 rpm steps. Fractions highly enriched in G2-phase cells were used for experiments.

### Sub-cellular Fractionation

For fractionation of proteins according to their intracellular localization, the Qproteome® Cell Compartment kit (Qiagen) was used following the procedures suggested by the manufacturer.

### Live Cell Imaging

To study intracellular localization of LIG3, DT40 cells expressing a LIG3-GFP fusion protein were directly stained for mitochondria visualization with 150 nM MitoTracker DeepRed (Invitrogen) for 1 h and for nuclei visualization with 1 µg/ml Hoechst 33342 (Invitrogen) for 30 min, all at 41°C. Immunofluorescence images of live cells were captured on a Leica TCS SP5 laser scanning confocal microscope using the LAS-AF software (Leica Microsystems) and were further processed using the Imaris software (Bitplane).

### *In vitro* Assay of NHEJ

Whole cell extracts were prepared using at least 30×10^6^ cells. Cells were collected, washed once with hypotonic buffer (5 mM KCl, 1.5 mM MgCl_2_, 0.2 mM phenylmethylsulfonyl fluoride (PMSF), 0.5 mM DTT and 10 mM HEPES-KOH, pH 7.5), resuspended in three packed-cell volumes of hypotonic buffer and subjected to three freeze–thaw cycles. Subsequently, KCl concentration was adjusted to 500 mM and the mixture incubated at 4°C for 30 min. The sample was cleared by centrifugation for 40 min at 14,000 rpm at 4°C, and the supernatant (whole cell extract) was dialyzed against dialysis buffer (25 mM HEPES-KOH, pH 7.5), 400 mM KCl, 1 mM EDTA, 10% glycerol, 0.2 mM PMSF and 0.5 mM DTT) overnight at 4°C [36]. Dialyzed cell extracts were cleared by a second centrifugation and snap frozen in small aliquots at –80°C.

In vitro end joining was measured with the pSP65 plasmid (3 kb, Promega) after linearization with *Sal* I [36]. End-joining reactions were performed in 20 mM HEPES-KOH, pH 7.5), 10 mM MgCl_2_, 80 mM KCl, 1 mM ATP, 1 mM DTT, 50 ng of substrate DNA and 0.5–2 µg of whole cell extract in a final volume of 20 µl at 25°C for 1 h. Reactions were terminated by adding 2 µl of 5% SDS, 2 µl of 0.5 M EDTA and 1 µg of protease (10 mg/ml) and incubating for 30 min at 37°C. One half of the reaction was loaded on a 0.7% agarose gel and run at 45 V (2 V/cm) for 4.5 h. Gels were stained with SYBR-Gold (Molecular Probes) and scanned using the Typhoon.

### Colony Formation Assay

Appropriate numbers of DT40 cells set in a manner allowing the accurate counting of the resulting colonies (typically 100–200) under the different conditions were seeded in medium containing 1.5% methylcellulose (MC) (Sigma) and incubated in 60 mm perti dishes for 10–14 d before counting.

### Purification of LIG3β

Recombinant human DNA ligase IIIβ was purified from bacteria as previously described [46].

### Ligase Activity Assay

For substrate preparation, an amount of 5 µg of oligo(dT)16 was radioactively labeled using 5 µl of γ-^32^P-ATP (Perkin Elmer NEG 502A, 3000 Ci/mmol) and 10 U of T4 polynucleotide kinase (Fermentas, EK0032). The reaction was incubated at 37°C for 30 min. Labeled oligos were purified on QIAquick® columns (Qiagen, Cat. no. 28304). The labeled oligonucleotides were mixed with an equimolar amount of polydA, incubated at 90°C for 10 min. and then slowly cooled to room temperature. Ligation reaction mixtures (30 µl) contained 60 mM Tris-HCl (pH 8.0), 10 mM MgCl_2_, 5 mM DTT, and 1 mM ATP, 50 µg/ml nuclease-free bovine serum albumin and 0,5–2,5 µg of whole-cell extract and were incubated at 16°C for 60 min. Reactions were stopped with 5 µl EDTA (1 M). An aliquot (5 µl) was heated for 5 min at 95°C in 65% formamide prior to loading onto a 10% acrylamide gel. After electrophoresis for 1.5 h at 300 V, gels were dried and analyzed by autoradiography. Ligation activity is expressed as percent of ligated substrate.

### Validation of LIG3 Knockout by PCR

Genomic DNA was isolated according to the NucleoSpin Tissue Kit (Macherey-Nagel) and DNA concentration was determined. PCR reactions were performed with 50 ng of DNA using Expand-Long-Template PCR System (Roche) according to the protocol of the manufactor. The primer sequences used were: 3LI34∶5′-TTAGCACCAGAATCAGACTTGGAGAGAAAT-3′ and 3LI32R: 5′-GCTACTTTTACTTAATTGCAGACATGAACC-3′ [18].

## Supporting Information

Figure S1 **(A)** Representative cell-cycle distribution histograms of wt DT40 cells incubated for 10 d in the presence or absence of 4HT. (**B)** Growth curves of wt DT40 cells grown in the presence or absence of 4HT. Cells were maintained in the exponential phase of growth by daily dilution in fresh growth medium. (**C)** Repair kinetics of IR-induced DSBs in asynchronous DT40 wt cells that were treated with 4HT, or were left untreated, after exposure to 40 Gy of X-rays. Results of at least three determinations from two independent experiments were used to calculate the indicated means and standard errors.
(TIF)

Figure S2 **(A)** Western blot analysis of LIG3 protein in *LIG3^−/2loxP^Cdc9* cells after treatment with 4HT for the indicated periods of time, or when left untreated. A mouse monoclonal antibody against human LIG3 (clone 1F3) that recognizes the chicken LIG3 was used. GAPDH is a loading control. **(B)** Western blot analysis of LIG3 and RAD51 proteins in wt DT40 cells treated with 10 µg/ml cycloheximide for the indicated periods of time. The treatment is toxic, interrupts cell growth and induces cell death starting at 4 h. Therefore results for up to 8 h of treatment are shown. (**C)** *In vitro* DNA end joining of *Sal*I-linearized *pSP65* plasmid using 1 µg whole cell extracts of the *LIG3^−/2loxP^Cdc9* mutant, prepared from untreated cultures, or cultures treated with 4HT for the indicated periods of time. The linearized input substrate plasmid (linear) and the products (dimers and multimers) generated by end joining are indicated. (**D)** *In vitro* DNA end joining of *Sal*I-linearized *pSP65* plasmid using increasing amounts of whole cell extracts prepared from wt and *LIG3^−/2loxP^* cells after treatment with 4HT for 2 d. The end joining activity loss of extracts prepared after 2 d treatment with 4HT can be rescued by the addition of 5 ng purified LIG3β, whereas end joining activity of extracts from wt cells is only slightly enhanced. The linearized input substrate plasmid (linear) and the products (circles, dimers and multimers) generated by end joining are indicated. (**E)** DNA ligase activity measured with oligo(dT)/poly(dA) substrates using whole cell extracts from *LIG3^−/2loxP^* cells at different times after incubation with 4HT. The graph shows the decrease in total DNA ligase activity, which in this case reflects the reduction in LIG3 levels. Results show the mean of three experiments.
(TIF)

Figure S3 (**A)** Growth kinetics of *LIG3^−/2loxP^LIG4^−/−^* cells and derivative clones 1, 3 and 7 expressing the mitochondrial version of hLIG1, *LIG3^−/2loxP^LIG4^−/−^mts-hLIG1.* Cells were maintained in the exponential phase of growth by daily dilution in fresh growth medium. (**B)** Human *LIG1* mRNA level measured by real-time PCR in clones 1, 3 and 7 of *LIG3^−/2loxP^LIG4^−/−^mts-hLIG1* cells normalized to that measured in clone 3. Results of independent determinations with two primer pairs were used to calculate the indicated means and standard errors. (**C)** Western blot analysis of LIG1 protein level in clones 1, 3 and 7 of the *LIG3^−/2loxP^LIG4^−/−^mts*-*hLIG1* mutant, of the *LIG3^−/2loxP^LIG4^−/−^* mutant, and of HeLa cells. A mouse monoclonal antibody recognizing human but not chicken LIG1 was used. GAPDH is used as loading control. (**D)** *LIG3* mRNA level measured by real-time PCR in clones 1, 3 and 7 of the *LIG3^−/2loxP^LIG4^−/−^mts-hLIG1* mutant and the parental *LIG3^−/2loxP^LIG4^−/−^* cells, normalized to the levels measured in wt cells.
(TIF)

Figure S4 **(A)** Growth kinetics of *Lig3^−/2loxP^Lig4^−/−^mts*-*hLig1* cells 5 d after 4HT treatment to convert to *Lig3^−/−^Lig4^−/−^mts*-*hLig1* cells. The growth of *Lig3^−/2loxP^Lig4^−/−^* cells immediately after treatment with 4HT is also shown for comparison. Cells were maintained in the exponential phase of growth by daily dilution in fresh growth medium. (**B)** Confirmation of excision of the *LIG3* exons between the loxP sites after treatment with 4HT, as measured by PCR using primers 3LI34 and 3LI32R (17) in clone 3 of the *LIG3^−/−^LIG4^−/−^mts*-*hLIG1* mutant. *LIG3^−/2loxP^LIG4^−/−^* and *LIG3^−/−^Cdc9* cells were used as controls. Primers are designed to bind before the first and in-between the two loxP sites on the conditional allele of *LIG3;* they produce a ∼3 kb product when the conditional allele is present and no product after 4HT treatment, when the segment between the two loxP sites is excised by Cre recombinase. PCR products have been fractionated on a 1% agarose gel and visualized by staining with EtBr. Product size was monitored with a DNA marker (GeneRuler™ 1 kb DNA Ladder, Fermentas). (**C)** *LIG3* mRNA level in clones 1, 3 and 7 of *LIG3^−/2loxP^LIG4^−/−^mts*-*hLIG1* cells after treatment with 4HT for 5 d to generate their *LIG3^−/−^LIG4^−/−^mts*-*hLIG1* cells. *LIG3^−/2loxP^LIG4^−/−^* cells are used as controls and mRNA levels are shown normalized to the wt. (**D)** Western blot analysis of Lig3 protein level in *LIG3^−/2loxP^LIG4^−/−^*, *LIG3^−/−^Cdc9* cells and clones 1, 3 and 7 of *LIG3^−/−^LIG4^−/−^mts*-*hLIG1* cells obtained after a 5 d incubation with 4HT. GAPDH is a loading control and purified human LIG3β a positive control. (**E)** Representative gels of *in vitro* DNA end joining of *Sal*I-linearized *pSP65* plasmid using whole cell extracts prepared from clones 1, 3 and 7 of the *LIG3^−/2loxP^LIG4^−/−^mts*-*hLIG1* mutant, before and after treatment with 4HT for 5 days. The linearized input substrate (linear) and the products of end joining (dimers and multimers) are indicated. Similar results were obtained using 2 µg of whole cell extract.
(TIF)

Table S1 **A summary of the key features of knockout, conditional knock-out and knock-in DT40 mutants generated and used in the present study.**
(PDF)
