# Supplementary figures and images for: DNA Ligases I and III Cooperate in Alternative Non-Homologous End-Joining in Vertebrates
Source: PLoS One. 2013 Mar 28;8(3):e59505. doi: 10.1371/journal.pone.0059505 (PMC3610672; doi:10.1371/journal.pone.0059505)

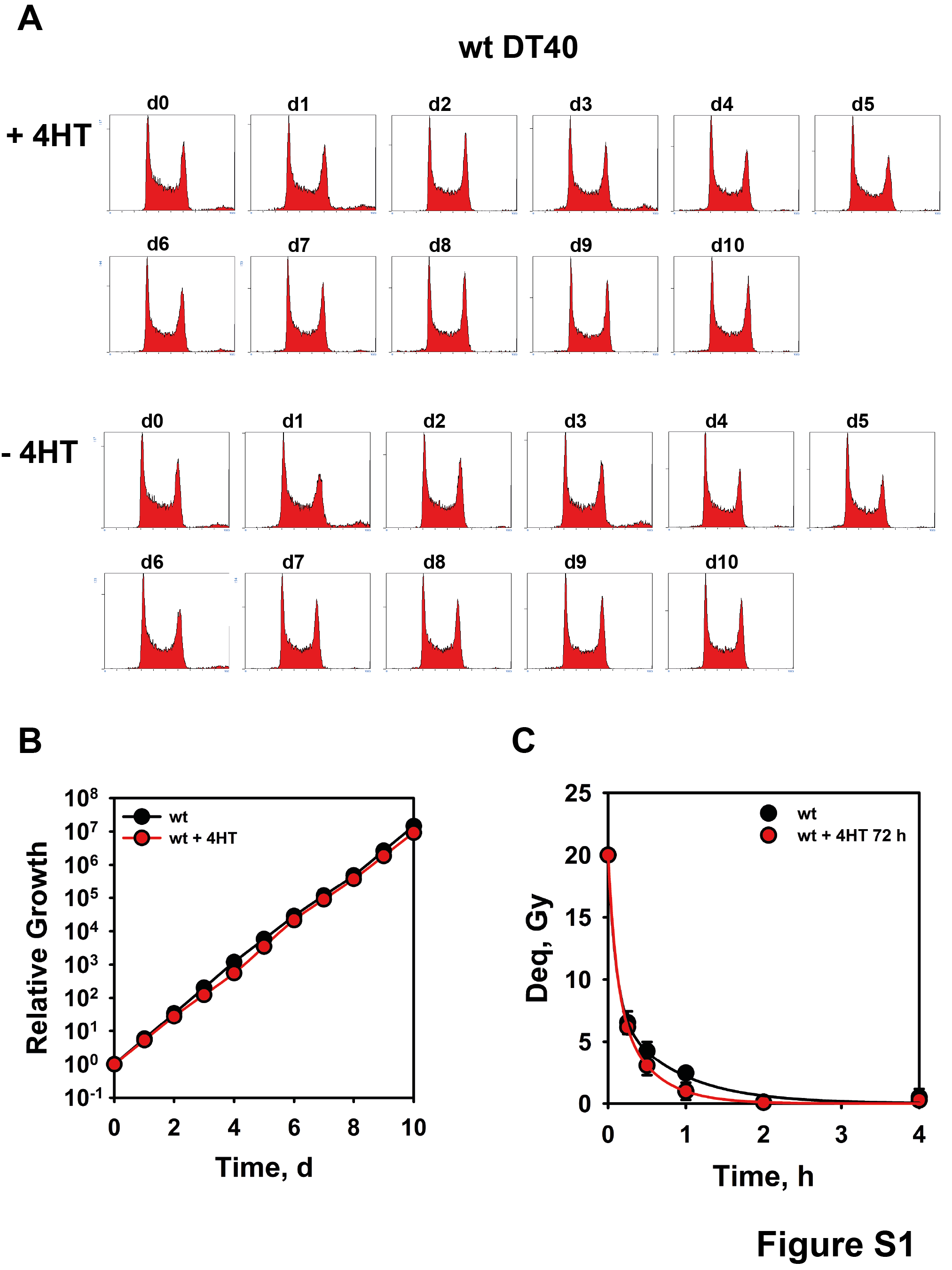

Supplement: Figure S1 — (A) Representative cell-cycle distribution histograms of wt DT40 cells incubated for 10 d in the presence or absence of 4HT. (B) Growth curves of wt DT40 cells grown in the presence or absence of 4HT. Cells were maintained in the exponential phase of growth by daily dilution in fresh growth medium. (C) Repair kinetics of IR-induced DSBs in asynchronous DT40 wt cells that were treated with 4HT, or were left untreated, after exposure to 40 Gy of X-rays. Results of at least three determinations from two independent experiments were used to calculate the indicated means and standard errors. (TIF) [file pone.0059505.s001.tif]

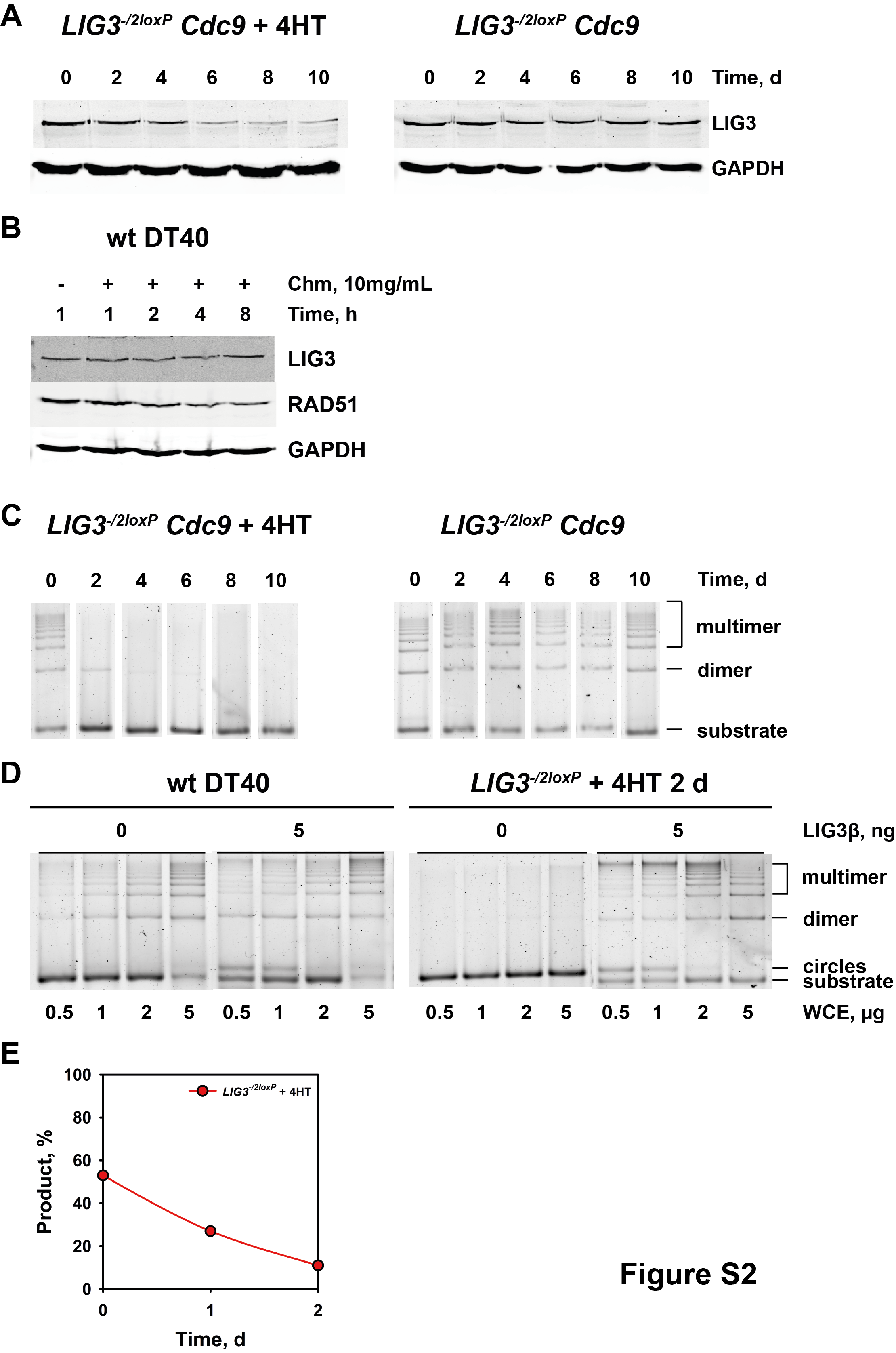

Supplement: Figure S2 — (A) Western blot analysis of LIG3 protein in LIG3−/2loxPCdc9 cells after treatment with 4HT for the indicated periods of time, or when left untreated. A mouse monoclonal antibody against human LIG3 (clone 1F3) that recognizes the chicken LIG3 was used. GAPDH is a loading control. (B) Western blot analysis of LIG3 and RAD51 proteins in wt DT40 cells treated with 10 µg/ml cycloheximide for the indicated periods of time. The treatment is toxic, interrupts cell growth and induces cell death starting at 4 h. Therefore results for up to 8 h of treatment are shown. (C) In vitro DNA end joining of SalI-linearized pSP65 plasmid using 1 µg whole cell extracts of the LIG3−/2loxPCdc9 mutant, prepared from untreated cultures, or cultures treated with 4HT for the indicated periods of time. The linearized input substrate plasmid (linear) and the products (dimers and multimers) generated by end joining are indicated. (D) In vitro DNA end joining of SalI-linearized pSP65 plasmid using increasing amounts of whole cell extracts prepared from wt and LIG3−/2loxP cells after treatment with 4HT for 2 d. The end joining activity loss of extracts prepared after 2 d treatment with 4HT can be rescued by the addition of 5 ng purified LIG3β, whereas end joining activity of extracts from wt cells is only slightly enhanced. The linearized input substrate plasmid (linear) and the products (circles, dimers and multimers) generated by end joining are indicated. (E) DNA ligase activity measured with oligo(dT)/poly(dA) substrates using whole cell extracts from LIG3−/2loxP cells at different times after incubation with 4HT. The graph shows the decrease in total DNA ligase activity, which in this case reflects the reduction in LIG3 levels. Results show the mean of three experiments. (TIF) [file pone.0059505.s002.tif]

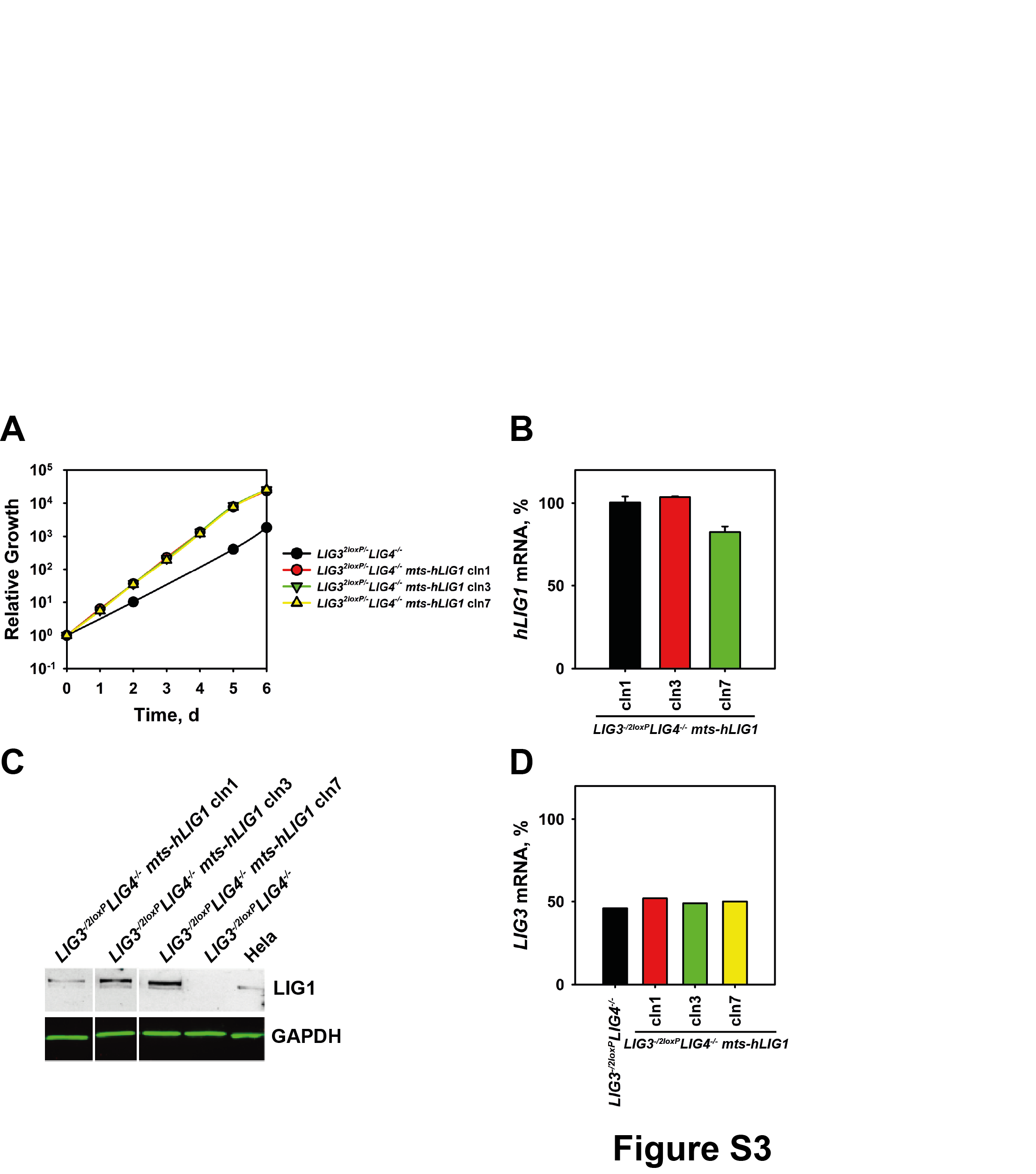

Supplement: Figure S3 — (A) Growth kinetics of LIG3−/2loxPLIG4−/− cells and derivative clones 1, 3 and 7 expressing the mitochondrial version of hLIG1, LIG3−/2loxPLIG4−/−mts-hLIG1. Cells were maintained in the exponential phase of growth by daily dilution in fresh growth medium. (B) Human LIG1 mRNA level measured by real-time PCR in clones 1, 3 and 7 of LIG3−/2loxPLIG4−/−mts-hLIG1 cells normalized to that measured in clone 3. Results of independent determinations with two primer pairs were used to calculate the indicated means and standard errors. (C) Western blot analysis of LIG1 protein level in clones 1, 3 and 7 of the LIG3−/2loxPLIG4−/−mts-hLIG1 mutant, of the LIG3−/2loxPLIG4−/− mutant, and of HeLa cells. A mouse monoclonal antibody recognizing human but not chicken LIG1 was used. GAPDH is used as loading control. (D) LIG3 mRNA level measured by real-time PCR in clones 1, 3 and 7 of the LIG3−/2loxPLIG4−/−mts-hLIG1 mutant and the parental LIG3−/2loxPLIG4−/− cells, normalized to the levels measured in wt cells. (TIF) [file pone.0059505.s003.tif]

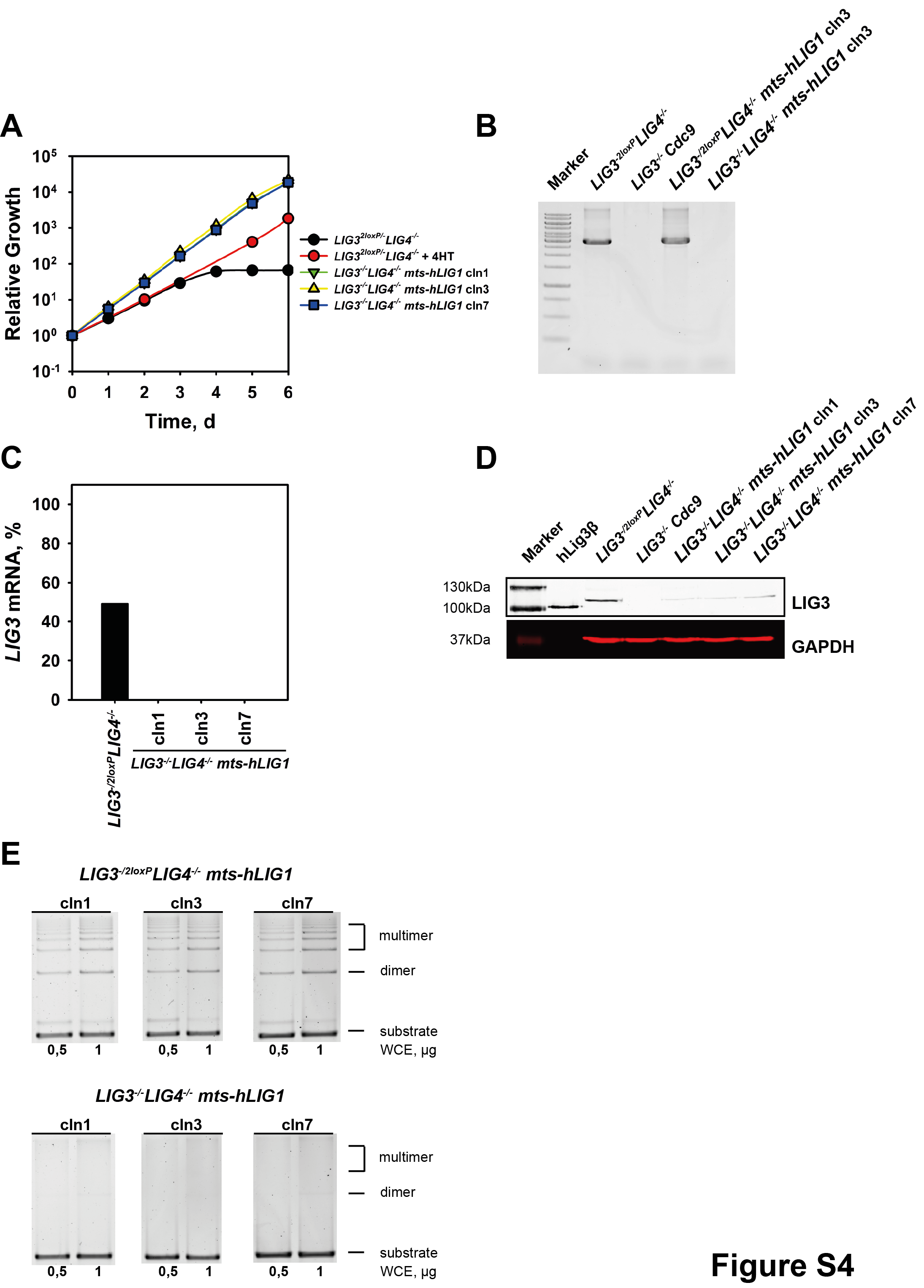

Supplement: Figure S4 — (A) Growth kinetics of Lig3−/2loxPLig4−/−mts-hLig1 cells 5 d after 4HT treatment to convert to Lig3−/−Lig4−/−mts-hLig1 cells. The growth of Lig3−/2loxPLig4−/− cells immediately after treatment with 4HT is also shown for comparison. Cells were maintained in the exponential phase of growth by daily dilution in fresh growth medium. (B) Confirmation of excision of the LIG3 exons between the loxP sites after treatment with 4HT, as measured by PCR using primers 3LI34 and 3LI32R (17) in clone 3 of the LIG3−/−LIG4−/−mts-hLIG1 mutant. LIG3−/2loxPLIG4−/− and LIG3−/−Cdc9 cells were used as controls. Primers are designed to bind before the first and in-between the two loxP sites on the conditional allele of LIG3; they produce a ∼3 kb product when the conditional allele is present and no product after 4HT treatment, when the segment between the two loxP sites is excised by Cre recombinase. PCR products have been fractionated on a 1% agarose gel and visualized by staining with EtBr. Product size was monitored with a DNA marker (GeneRuler™ 1 kb DNA Ladder, Fermentas). (C) LIG3 mRNA level in clones 1, 3 and 7 of LIG3−/2loxPLIG4−/−mts-hLIG1 cells after treatment with 4HT for 5 d to generate their LIG3−/−LIG4−/−mts-hLIG1 cells. LIG3−/2loxPLIG4−/− cells are used as controls and mRNA levels are shown normalized to the wt. (D) Western blot analysis of Lig3 protein level in LIG3−/2loxPLIG4−/−, LIG3−/−Cdc9 cells and clones 1, 3 and 7 of LIG3−/−LIG4−/−mts-hLIG1 cells obtained after a 5 d incubation with 4HT. GAPDH is a loading control and purified human LIG3β a positive control. (E) Representative gels of in vitro DNA end joining of SalI-linearized pSP65 plasmid using whole cell extracts prepared from clones 1, 3 and 7 of the LIG3−/2loxPLIG4−/−mts-hLIG1 mutant, before and after treatment with 4HT for 5 days. The linearized input substrate (linear) and the products of end joining (dimers and multimers) are indicated. Similar results were obtained using 2 µg of whole cell extract. (TIF) [file pone.0059505.s004.tif]
