# Supplementary material for: DNA Ligases I and III Cooperate in Alternative Non-Homologous End-Joining in Vertebrates
Source: PLoS One. 2013 Mar 28;8(3):e59505. doi: 10.1371/journal.pone.0059505 (PMC3610672; doi:10.1371/journal.pone.0059505)
Supplement: Table S1 — A summary of the key features of knockout, conditional knock-out and knock-in DT40 mutants generated and used in the present study. (PDF) [file pone.0059505.s005.pdf]

Table S1

Cell line	Feature
LIG1 $^{-/-}$	*LIG1* knockout
LIG3 $^{-/2loxP}$	*LIG3* conditional knockout
LIG3 $^{-/-}$ *****	*LIG3* knockout
LIG3 $^{-/M2I}$	nuclear *LIG3* knockout
LIG4 $^{-/-}$	*LIG4* knockout
LIG1 $^{-/-}$ ***LIG4*** $^{-/-}$	*LIG1* knockout; *LIG4* knockout
LIG3 $^{-/2loxP}$ ***LIG4*** $^{-/-}$	*LIG3* conditional knockout; *LIG4* knockout
LIG3 $^{-/-}$ ***LIG4*** $^{-/-}$ *****	*LIG3* knockout; *LIG4* knockout
LIG3 $^{-/M2I}$ ***LIG4*** $^{-/-}$	nuclear *LIG3* knockout; *LIG4* knockout
LIG3 $^{-/-}$ ***Cdc9***	*LIG3* knockout overexpressing *CDC9* (yeast *LIG1*)
LIG3 $^{-/2loxP}$ ***hLIG3α***	*LIG3* conditional knockout overexpressing human nuclear *LIG3α*
LIG3 $^{-/-}$ ***hLIG3α*** *****	*LIG3* knockout overexpressing human nuclear *LIG3α*
LIG3 $^{-/2loxP}$ ***LIG4*** $^{-/-}$ ***mts-hLIG1***	*LIG3* conditional knockout; *LIG4* knockout overexpressing human mitochondrial *LIG1*
LIG3 $^{-/-}$ ***LIG4*** $^{-/-}$ ***mts-hLIG1*** *****	*LIG3* knockout; *LIG4* knockout overexpressing human mitochondrial *LIG1*

***** Conditional allele deleted by 4HT treatment of parental cell line.
